# Cellular and molecular mechanisms of aspartoacylase and its role in Canavan disease

**DOI:** 10.1186/s13578-024-01224-6

**Published:** 2024-04-06

**Authors:** Martin Grønbæk-Thygesen, Rasmus Hartmann-Petersen

**Affiliations:** The Linderstrøm-Lang Centre for Protein Science, Department of Biology, University of Copenhagen, Ole Maaløes Vej 5, 2200N Copenhagen, Denmark

**Keywords:** Protein folding, Protein stability, Protein degradation, Protein quality control, Protein misfolding, Proteasome, VUS, Neurodegeneration, NAA, NAAG

## Abstract

Canavan disease is an autosomal recessive and lethal neurological disorder, characterized by the spongy degeneration of the white matter in the brain. The disease is caused by a deficiency of the cytosolic aspartoacylase (ASPA) enzyme, which catalyzes the hydrolysis of N-acetyl-aspartate (NAA), an abundant brain metabolite, into aspartate and acetate. On the physiological level, the mechanism of pathogenicity remains somewhat obscure, with multiple, not mutually exclusive, suggested hypotheses. At the molecular level, recent studies have shown that most disease linked *ASPA* gene variants lead to a structural destabilization and subsequent proteasomal degradation of the ASPA protein variants, and accordingly Canavan disease should in general be considered a protein misfolding disorder. Here, we comprehensively summarize the molecular and cell biology of ASPA, with a particular focus on disease-linked gene variants and the pathophysiology of Canavan disease. We highlight the importance of high-throughput technologies and computational prediction tools for making genotype–phenotype predictions as we await the results of ongoing trials with gene therapy for Canavan disease.

## Introduction

Aspartoacylase (EC 3.5.1.15; UniProt ID: P45381) (ASPA) – also known as aminoacylase II (ACY2) [1] or N-acetyl-L-aspartate amidohydrolase [2] in older literature, is a 35.7 kDa, 313 residue enzyme that catalyzes the hydrolysis of N-acetyl-L-aspartic acid (NAA) into acetate and aspartate [3–5]. In humans, the *ASPA* gene is located on the short arm of chromosome 17, spans 29 kb and contains 6 exons [3, 6]. The ASPA mRNA is widely distributed, but particular expressed in kidney and in oligodendrocytes of the brain [7–9]. The encoded enzyme is a single domain homo-dimeric protein complex, with a highly specific active site buried within a channel in the native protein [10]. Insufficient ASPA activity caused by germline *ASPA* variants is linked to Canavan disease (CD) (OMIM: 271900), a recessive, neurodegenerative leukodystrophy, where oligodendrocytes fail to correctly myelinate neuroaxons [11]. While the precise pathogenic mechanism remains elusive, several non-mutually exclusive hypotheses have been proposed [12–15].

Clinically, CD patients display poor muscle control, decreased cognitive capabilities and other severe conditions. Typically, these symptoms appear early, already within the first 3–6 months of life, and gradually progress over time eventually leading to an early death [16]. Various attempts at ameliorating the symptoms [17–19] and curing the disease [20–22] have been reported, including more recently promising attempts and ongoing trials with gene therapy [21, 23, 24]. Accordingly, clinical classification and a detailed understanding of how pathogenic *ASPA* gene variants operate are highly warranted. Using deep mutational scanning technologies, it was recently shown that most loss-of-function missense variants cause a structural destabilization of the ASPA protein structure, leading to the formation of non-native ASPA proteins that negatively affect cell fitness and are subject to rapid degradation by the cellular protein quality control (PQC) system [25]. Accordingly, studies on ASPA, therefore also provide a model system for understanding intracellular protein folding, misfolding and the PQC system, which ultimately may further our understanding of CD and other protein misfolding diseases.

Here we comprehensively summarize the physiological, cellular, and molecular details of the ASPA enzyme, its substrate NAA and Canavan disease. We focus particularly on the pathogenic *ASPA* gene variants, the importance of variant classification and gaining a mechanistic understanding of how loss of function gene variants operate for future implementation of gene therapy or other forms of precision medicine.

### *ASPA* gene expression

While *ASPA* gene expression is elevated in the brain and even higher in the kidneys [1, 2, 7, 26–29], the protein also seems to be present to a lesser extent in several other tissues, including liver, intestine and lung [1, 7, 30–32]. Indeed, skin fibroblasts have also been used to obtain ASPA for activity assays [32–34].

However, the most important site of *ASPA* expression is the brain white matter (WM), where the enzyme plays an essential role in NAA catabolism, as evident by the following investigations with rats. Some studies, using antibodies, detected ASPA protein clearly in oligodendrocytes, and faintly in neurons and microglial-like cells, but not in astrocytes [35, 36]. These observations were corroborated by two other studies of both ASPA mRNA [8] and protein [37], which found it to be restricted [37] or primarily restricted [8] to oligodendrocytes. Similarly, one study detected ASPA enzymatic activity in oligodendrocytes, but not astrocytes [38]. However, yet another study found ASPA activity in O2A progenitor cells and both their differentiated cell types (astrocytes and oligodendrocytes), but not in neurons [39]. It has been reported that rat Schwann cells (the peripheral nervous system equivalent of oligodendrocytes) do not express ASPA mRNA [8], while a later study in mice found Schwann cells to express ASPA. However, the authors noted that the peripheral nerves looked grossly normal in CD mice, thus emphasizing the role of ASPA primarily within the central nervous system (CNS) [30]. This was corroborated by a study of the auditory processing in the same mouse strain, showing functional and morphological deterioration was limited to the CNS, with the cochlear nerve fibers being unaffected [40].

Regardless of these discrepancies, there seems to be consensus that ASPA is mainly associated with oligodendrocytes and their myelin sheaths. A notion supported by the fact that oligodendrocyte-specific *ASPA* knock-out in mice causes the same – albeit milder – phenotype in the CNS as whole body *ASPA* knock-out [41]. Further supporting this idea, is the low ASPA activity observed in grey matter (GM) [39], and the fact that the brain periphery remains unaffected in CD patients [29]. Even within the brain WM, ASPA expression exhibits spatiotemporal regulation [35, 39]. Temporally, little to no ASPA seems to be present in neonatal rats, with levels starting to rise postnatally, coinciding with the myelination of the brain for it to then decrease somewhat while maintaining a detectable level in adult rats [8, 37, 39, 42].

### ASPA protein structure

**Fig. 1:**
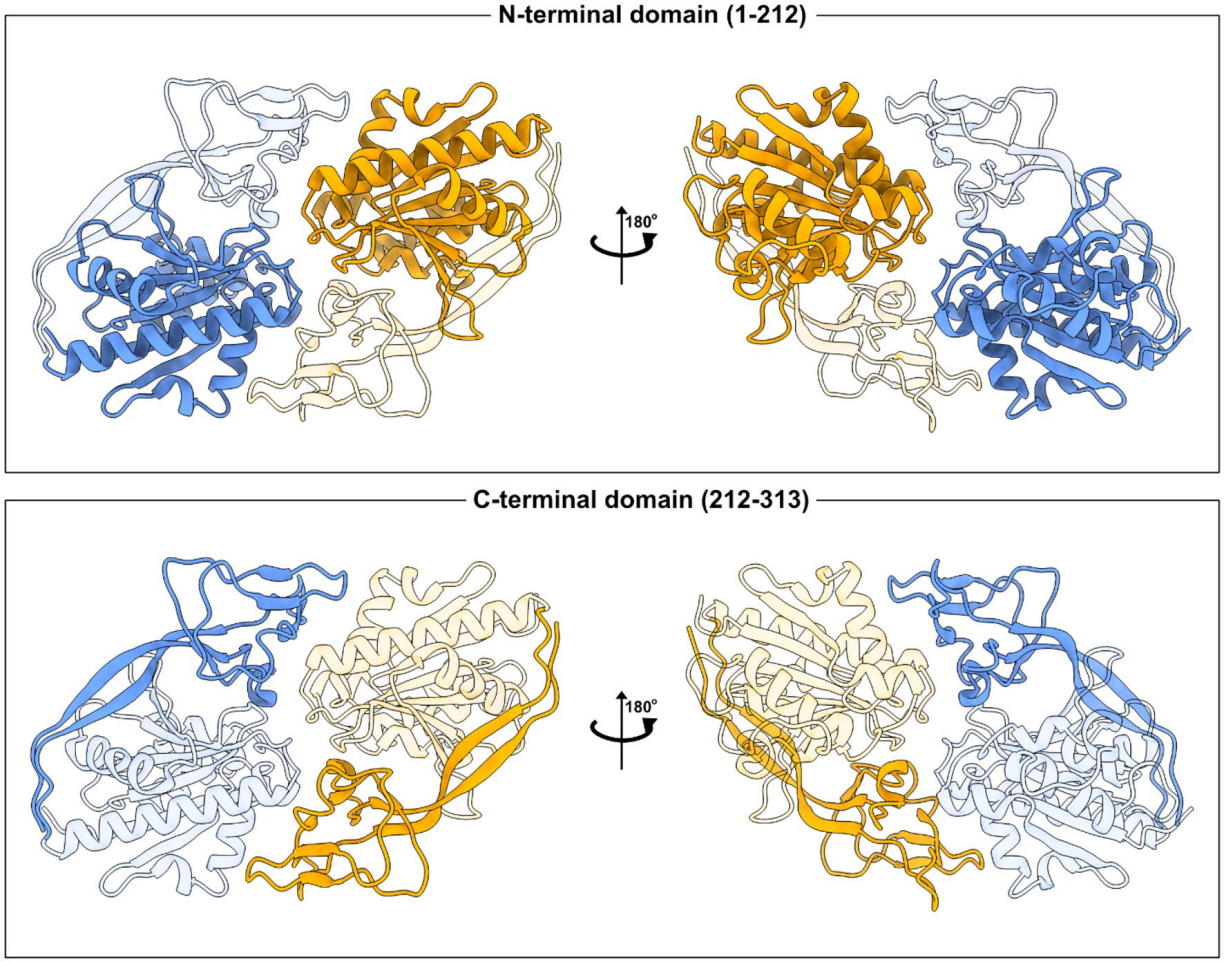
*The ASPA protein structure*. The structure of the human ASPA homodimer (PDB: 2O53) [44] is shown with the two subunits in blue and yellow. The N-terminal region covering residues 1–212 (upper panel) and C-terminal region spanning residues 212–313 (lower panel) are highlighted

ASPA consists of an N-terminal (residues 1–212) and a C-terminal domain (resides 213–313) (Fig. 1). The N-terminal region consists of a central six-stranded mixed β-sheet surrounded by eight helices of variable size and multiple connecting loops. The C-terminal domain consists of two antiparallel β-sheets that wrap around the substrate-binding side of the N-terminal domain with a globular portion between the two β-sheets [10]. The two domains connect though various interactions, including a β-sheet anchor formed between β1 and β13 at the very N- and C-terminal ends of the domain (based on the solved structure of rat ASPA and the AlphaFold predictions for human ASPA). Additionally, the C-terminal region of ASPA wraps around the N-terminal region via the antiparallel β-strands β8 and β12 [10]. Consequently, the N-terminal domain is not stable when expressed on its own [25, 43], and the two domains should thus be considered as one joint unit. Together, the N- and C-terminal regions form a channel leading to the active site.

Sequence alignments [45] and structural analyses [10, 46] have demonstrated similarities between the N-terminal part of ASPA and a range of Zn^2+^-dependent carboxypeptidase A-related hydrolases [10, 45]. However, carboxypeptidase A has a ~ 60 residue N-terminal extension of the central β-sheet by two strands and carboxypeptidases completely lack the C-terminal extension [10]. More specifically, ASPA belongs to the succinyl glutamate desuccinylase/aspartoacylase family (AstE/AspA, PFAM04952) [10]. The C-terminal region in ASPA likely reflects a requirement for high substrate specificity for ASPA, which is localized in the cytosol, compared to carboxypeptidase A, which cleaves a range of peptides in the small intestine.

Unlike aminoacylase I, which hydrolyses N-acetyl groups from all amino acids, ASPA (also known as aminoacylase II) exhibits high substrate specificity towards N-acetyl-L-aspartic acid [16, 47].

**Fig. 2:**
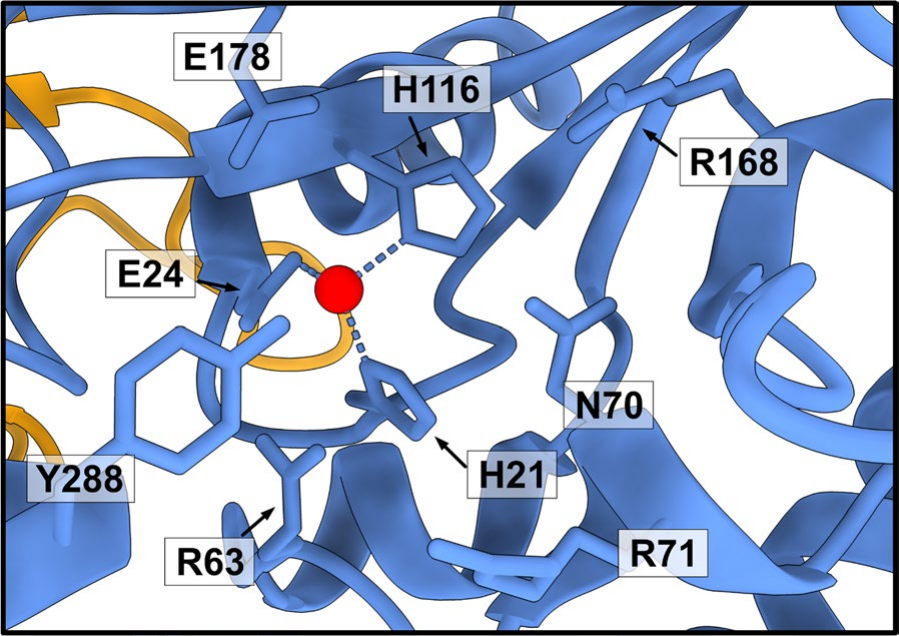
*The active site*. A zoom in on the active site within one subunit of the human ASPA structure (PDB: 2O53) [44] and residues (H21, E24, H116) coordinating the Zn^2+^ ion (red). Residues Arg63, Asn70, Arg71, Arg168, and Tyr288 interact with the substrate

The C-terminal extension of ASPA shields the active site, restricting access to it. In the entry channel R71, K228, K291, K292 and E293 provide a positive electrostatic potential, which may guide NAA to the active site while repelling positively charged metabolites. Although some peptides may enter the channel, the C-terminal region would orientate them in a position that does not enable hydrolysis to occur [10, 48]. A tight pocket consisting of residues T118, Q184, F282, E285, A287, and Y288 accommodates the acetyl group of NAA, while restricting compounds with acyl groups longer than acetate. ASPA also shows high selectivity towards the aspartate-side of NAA [49], possible due to a hydrogen bond between R168 and the β-carbonyl group of NAA [10]. The key catalytic core residues include: R63, N70, R71, R168, E178 and Y288 as well as H21, E24 and H116, which coordinate the catalytic Zn^2+^ ion (Fig. 2).

**Fig. 3:**
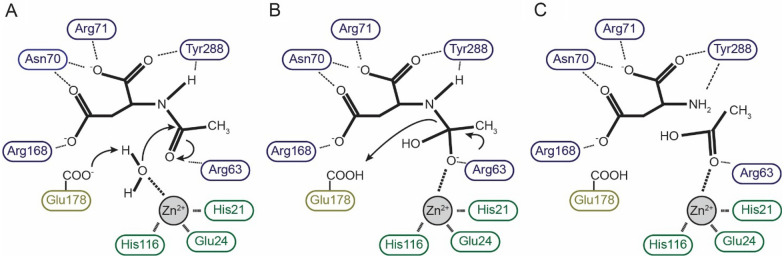
*Overview of the proposed catalytic mechanism of aspartoacylase*. **A** First, water initiates a nucleophile attack on the NAA carbonyl group leading formation of **B** a tetrahedral intermediate and finally **C** the products. Green indicates residues coordinating Zn^2+^, purple indicates residues interacting with the substrate, and yellow indicates the catalytic active E178 residue

A “promoted-water pathway” mechanism similar to that of carboxypeptidase A, has been proposed for the hydrolysis catalyzed by ASPA **(**Fig. 3). In this model, E178 deprotonates a water molecule, which is stabilized by Zn^2+^. The resulting hydroxide then attacks the β-carbonyl group of NAA, which is stabilized by R63 and possibly also the Zn^2+^ ion (Fig. 3A), to allow the formation of a tetrahedral intermediate (Fig. 3B). Lastly, the intermediate collapses with aspartate being eliminated as the leaving group [10] (Fig. 3C). The model is supported by computational analyses, which also indicated that substrate release, rather than bond cleavage, is the rate limiting step of the reaction [50].

### Homodimer formation and its functional implications

Solved crystal structures of human and rat ASPA shows that they form similar homodimers. In humans, the dimer interface covers ~ 1200 Å^2^ of surface accessible solvent area and involves 12 hydrogen bonds and two salt bridges [10, 44]. This structural evidence of dimerization has been supported by various biochemical assays performed on human and rat ASPA [10, 37, 46, 49]. However, some of these observations could be explained by aggregation [49] or antibody cross-reactivity [26, 37]. Additionally, size-exclusion chromatography showed the ASPA monomer is enzymatically active, but does not exclude the possibility of an ASPA homodimer [26]. Hence, despite the data supporting an ASPA homodimer, the importance of the dimer formation remains somewhat elusive, but could allow for allosteric and cooperative regulation of the enzyme.

In agreement with this, human ASPA produced from *Pichia pastoris* has been shown to display unusual enzymatic properties. Thus, at low NAA levels, the enzyme showed sigmoidal behavior indicative of subunit cooperativity. Conversely, at high NAA levels significant substrate inhibition was observed, indicative of non-competitive inhibition of ASPA through non-catalytic NAA-binding sites on ASPA. Notably, similar behavior was observed when using the alternative substrate N-trifluoroacetyl-L-aspartate (trifluoro-NAA) [46].

Molecular dynamics (MD) simulations and molecular docking studies of ASPA have attempted to elucidate some of the above observations, revealing that monomeric ASPA exists predominantly in a “closed” conformation where access to the active site is hindered by the “gate residues” mainly constituted by the R71-E293 salt bridge and the hydrogen bonded pair Y64-K291. However, in the dimeric state, one monomer remains in the “closed” conformation, whereas the other one fluctuates between the “open” and “closed” conformations, allowing substrate entry to the active site. Additionally, an activating allosteric NAA binding site was observed for each monomer, as well as an inhibitory binding site near the dimer interface. The activating site includes residues R56, K59, and K60, is easily accessible and has a predicted binding free energy of -6.5 kcal/mol, whereas the inhibitory site, involves the side chains of K292 and R233 as well as the main chains of E290 and G237, is more secluded in the structure, and has a predicted binding energy of − 4.7 to − 4.8 kcal/mol [48, 51]. Despite, the two monomers in the dimers being close to identical [44], the simulations found their dynamic properties to differ, which could explain the existence of one shared inhibiting NAA allosteric site between the two subunits, and why one subunit remains inactive [48]. The positive allosteric site explains the observed sigmoidal behavior [46] at low NAA levels [51]. Conversely, at high NAA levels, binding to the inhibitory allosteric site of one monomer affects the dimer interface communication, leading to a pathway of conformational changes that propagate through the other monomer. This increases the rigidity of the loops with the gate-forming residues in loops 62 − 74 and 282 − 294, thus hindering access to the active site [48]. Yet the biological rationale behind the negative allosteric regulation remains enigmatic.

Considering the allosteric nature of ASPA, where dimer interactions are necessary for efficient catalysis, the risk of dominant negative effects, where a non-functional ASPA variant dimerizes and inhibits the wild-type subunit, seems plausible. However, given the recessive nature of the disease, this is not the case. The catalytic requirement is potentially low, meaning that in a heterozygous individual, the formed wild-type/wild-type dimers are sufficient, even if the other ASPA variant is inactive and forms inactive wild-type/variant dimers. Moreover, the rapid degradation of many disease-linked variants [25, 52] also increases the likelihood of forming wild-type dimers in heterozygous cells. Additionally, dimer formation may preferentially be allele-specific since newly synthetized ASPA monomers from the same mRNA are more likely to form dimers. This has been proposed as a general mechanism that buffers proteins against dominant negative effects [53]. Indeed, the large interaction surface between the subunits in the ASPA dimer, suggests that the dimer is stable. In turn, this reduces the risk of forming dominantly negative inactive wild-type/variant dimers. Since the dimer-interface involves both the N- and C-terminal regions [10, 44], co-translational assembly [54] would likely require that the N-terminal part of ASPA co-translationally binds the C-terminal region of a pre-existing or newly synthetized ASPA monomer.

The contributions from allosteric regulation and stabilization from homodimers, makes predictions of variant effects harder, and poses a challenge for genotype–phenotype predictions of CD. Yet, to the extent it occurs in ASPA, cis-assembly of allele specific monomers, will limit these effects.

### ASPA enzyme activity assays

**Fig. 4:**
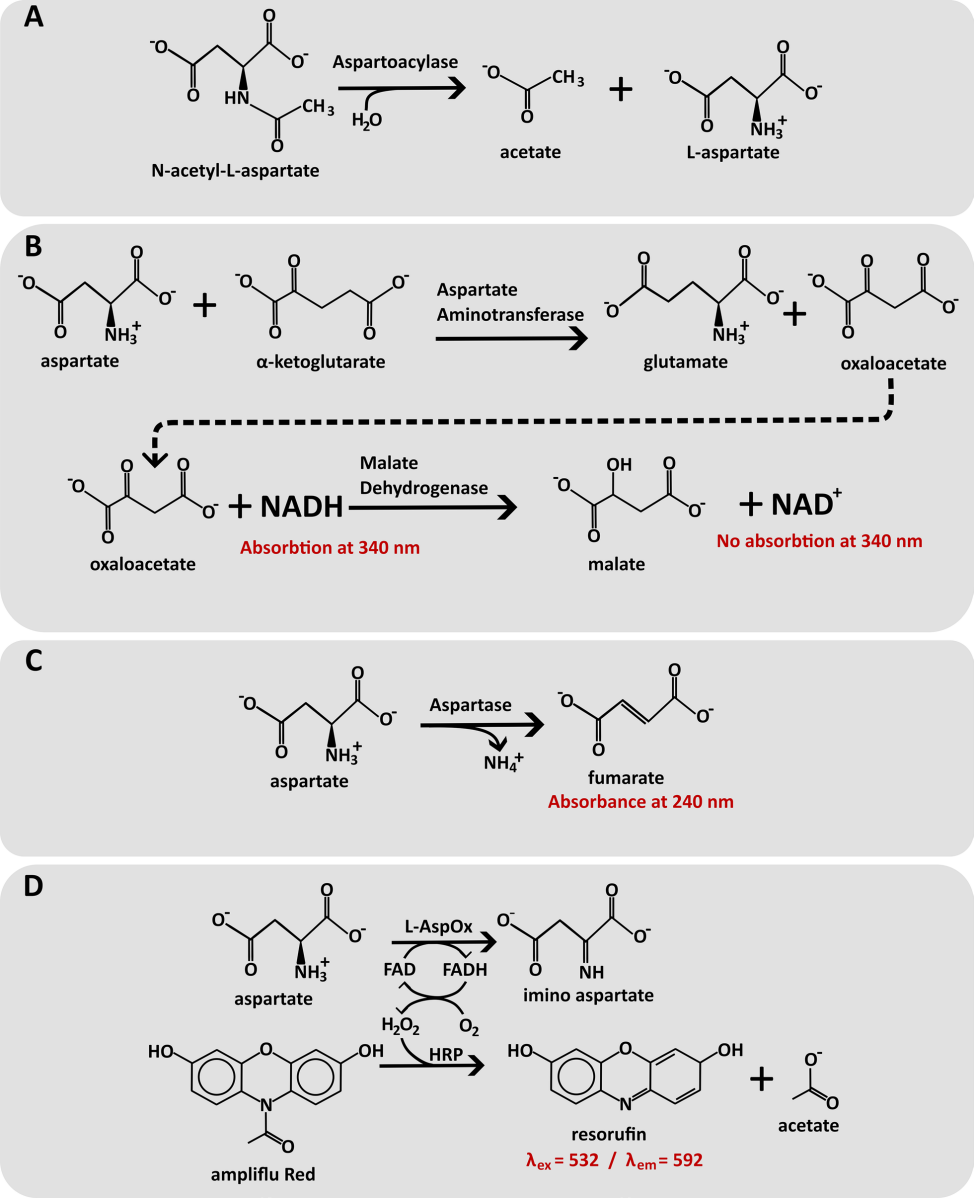
*Overview of the enzymatic reactions used to assay ASPA activity.* **A** ASPA hydrolyzes N-acetyl-L-aspartate (NAA) to acetate and aspartate. The released aspartate is coupled to other enzymatic reactions, such as **B** aspartate aminotransferase and malate dehydrogenase [32], **C** aspartase [49], and **D** aspartate oxidase [58]. The reactions can be followed by spectrophotometry based on the indicated specifications (red)

With the obvious diagnostic value for Canavan disease, much work has been put into assays to determine ASPA enzymatic activity. One assay utilizes coupling of NAA hydrolysis to NADH oxidation (Fig. 4AB). Here, α-ketoglutarate and aspartate aminotransferase is used to create glutamic acid and oxaloacetate from the aspartate product. In the second reaction, malate dehydrogenase reduces oxaloacetate into malate, simultaneously oxidizing NADH into NAD^+^. Since only the reduced NADH-form has a noticeable absorbance at 340 nm, NAA hydrolysis can be detected as a drop in absorbance at 340 nm [55]. This assay has been used for many decades [1, 32], with different modifications [39, 56, 57]. A slightly more simple method uses aspartase to convert aspartate into fumarate, which absorbs light at 240 nm [49] (Fig. 4AC).

Some groups have utilized NAA with ^14^C-labelled aspartate and thin-layer chromatography to assess NAA hydrolysis [26, 43, 59], while others have examined residual NAA using high-pressure liquid chromatography (HPLC) [38, 60]. Somewhat similarly, ASPA activity has been measured as the release of tritium-labelled acetate from ^3^H-NAA [33, 61]. Furthermore, the distinct peaks in the ^1^H NMR spectra of NAA and its product acetate, provides another means of measuring ASPA activity, even without the use of isotope-label NAA [62].

Recently, a new activity assay was developed, where aspartate is oxidized using L-aspartate oxidase (Fig. 4AD). This creates H_2_O_2_ which can be measured with peroxidase in a fluorimetric assay. While inferior to chromatography-based assays at low NAA concentration samples, the assay is scalable, potentially allowing for high-throughput determination of ASPA enzyme activities [58].

An alternative approach for a high-throughput ASPA activity assay, would be a yeast survival assay, where yeast—complimented with a library of ASPA variants – is grown in a minimal media where hydrolysis of NAA by ASPA constitutes the only nitrogen or carbon source. However, when attempted, using WT *S. cerevisiae* transformed with vector or WT ASPA, no differences in growth were observed, with either variant growing in carbon-deficient media and both growing equally well in nitrogen-deficient media [52].

Estimates of the specific activity of ASPA vary across the literature, likely due to variations between measuring techniques, protein purification protocols, and buffer conditions. For example, it has been reported that ASPA activity doubled when a phosphate buffer was exchanged for a Tris buffer [56]. In addition, human ASPA appears more stable and active when purified from *P. pastoris* instead of *E. coli* [46]. Hence, comparing specific ASPA activities across publications is of limited use. However, enzyme activities of ASPA variants relative to wildtype ASPA can be estimated [7, 43, 60, 63, 64], although the rather poor enzymatic activity of ASPA remains a hurdle for obtaining useful measurements. Examples of reported specific activities of ASPA are listed in Table 1. It is tempting to attribute the low enzyme activity to the inhibitory effect of excess NAA [46], but it is also likely a consequence of the high substrate-specificity [10], and ASPA activity is thus limited by the speed at which products can leave the entry channel [50].

**Table 1:** Reported specific activities

Reported activity	Assay	Source	References
1185 μmol/h/mg ASPA	Malate dehydrogenase	Bovine brain	[57]
3 μmol/h/mg ASPA	Aspartase-coupled	*E. coli*	[49]^a^
~ 3 μmol/h/mg ASPA	TLC	Rat kidney	[26]^b^
600–900 μmol/h/mg ASPA	Aspartase-coupled	*P. pastoris*	[46]^c^
300 μmol/h/mg ASPA	HPLC	*E. coli*	[60]^b^
450 μmol/h/mg ASPA	Aspartase-coupled	*P. pastoris*	[63]^c^

^a^Same specific activity was observed for mouse and human ASPA.

^b^Activities were obtained as estimates from figure graphs.

^c^No unit definition included; we assumed 1 U to be 1 μmol/min

### Canavan disease

Defects in ASPA functionality leads to Canavan disease (MIM# 271900), a type of leukodystrophy [11]*.* The initial clinical characterization of the disease is accredited a publication by Myertelle Canavan from 1931 [65], who described spongyform degeneration of brain WM in what she initially diagnosed to be a case of Schidler’s disease (another leukodystrophic disease) in a child [16]. Similar brain pathology was described in an earlier report by Globus and Strauss [66], but also diagnosed as Schidler’s disease [67]. In 1949 the phenotype was recognized as a distinct disease by Van Bogaert and Bertrand, who also reported its autosomal recessive inheritance pattern and high prevalence among the Ashkenazi Jewish population [16, 67, 68]. Hence, the disease has also occasionally been referred to as the”Canavan-Van Bogaert-Bertrand” disease [19].

The enzyme was initially purified from porcine kidney [1, 4, 5, 26], but was not linked to Canavan disease until a few decades later. The major breakthrough in the understanding of Canavan disease came with two discoveries. Firstly, elevated NAA levels in the urine (N-acetylaspartic aciduria) was observed for a child with extensive and progressive cerebral atrophy by Kvittingen et al. [69]. Secondly, Hagenfeldt et al. observed similar leukodystrophic symptoms and N-acetylaspartic aciduria in another child, and linked it to ASPA deficiency [32]. Soon thereafter, the symptoms of the two patients and others were recognized to be similar to those described by Van Bogaert and Bertrand [56, 70]. Thus, Canavan disease was characterized as a monogenic disease, caused by insufficient aspartoacylase activity leading to loss of NAA catabolism [71, 72]. Later significant milestones include the cloning of human *ASPA* cDNA in 1993 [3], and discovery of the first specific mutations in *ASPA* [73].

### Symptoms of Canavan disease

Symptoms of CD typically manifest within the first 3–6 [16] or 0–6 [74] months based on different reports. Common, early symptoms include megalencephaly (enlargement of the head), hypotonia (loss of muscle tone), developmental delays, increased irritability and abnormal eye movements / nystagmus [16, 74]. The triad of hypotonia, head lag and megalencephaly should suggest Canavan disease, when WM involvement is suspected [16]. Past the first 4–6 months of life, the developmental delays and megalencephaly becomes more apparent [16, 74, 75]. Psychomotor development in patients is usually limited to that of a 1-year-old, with few patients acquiring fine motor skills such as the ability to draw or scribble. Likewise, only 3 out of 23 patients in one study were able to speak single words, and none could form complete sentences [74]. With age, hypotonia develops into spasticity [16, 74] and the developmental delays become more apparent [67]. Especially motor and verbal skills are affected, with most Canavan disease children being unable to properly sit, stand, walk or talk [67]. However, in spite of profound delays, CD patients can sometimes interact with others, smile, and reach for objects [75]. In addition to hypotonia, contractures and decubiti has also been reported for some CD patients, which needs to be prevented by exercise and position changes [76]. In these cases, physical therapy is recommended to minimize contractures and maximize motor abilities and seating posture [76, 77]. Additional symptoms may include feeding difficulties, sleep disturbances, and poor vision [16, 74]. Many patients may require assisted feeding through a gastric tube [67, 74, 78] or permanent gastronomy [67], as their ability to swallow voluntarily is lost [75]. Approximately 57% of CD patients also develop seizures [74, 78, 79] often requiring anticonvulsant medication [74]. A newer study found that, while rare in the first year of life, seizures increase in frequency over time in most patients, with the highest frequency towards the end of the first decade of life [74]. Another study put the mean onset of seizures at 9 months of age, also noting that the seizures were generalized tonic–clonic (*i.e.* involving both tonic (stiffening) and clonic (twitching or jerking) phases of muscle activity) [80].

Previously, many patients succumbed to the disease within the first years of life. However, improved medical and nursing care has extended life expectancy, with a significant number of patients now reaching their second or even third decade of life [16, 74, 78, 81, 82].

#### Macroscopic and histopathological symptoms

Macroscopically, the brain weight of CD patients under 20 months of age is 150% above normal average, whereas for patients over 30 months it had normalized to 103%. This is most likely attributed to macroencephaly and subsequent brain degeneration, respectively [83]. An ill-defined demarcation of cortical GM and WM has also been observed, likely caused by the poor myelination [83, 84].

Histologically, Canavan disease is characterized by progressive spongiform degeneration of the brain WM [71, 84], where oligodendrocytes, found within the brain WM, fail to myelinate the neuroaxons, thus rendering the neurons unable to function normally [11]. Vacuoles were observed within the myelin sheaths in the subcortical WM [71]. Moreover, a significant number of Alzheimer type II astrocytes were found in the cerebral cortex, cerebellum, and basal ganglia [71, 83], although this was not observed for one investigation, which reported only a few scattered Alzheimer type II astrocytes in both WM and GM [85].

Interestingly, a study reported that neurons appeared normal [83], although mouse studies on ASPA deficient mice have shown vacuolization and axonal loss in the cerebellum, suggesting some extent of neuronal damage does occur [86, 87]. Hypertrophy, hyperplasia [88, 89] and astrocytic gliosis (astrocytosis) [90, 91] have also been reported, with some cells having unusually elongated mitochondria [88, 89, 92].

### Different subtypes of Canavan disease

It is apparent that the severity of CD patients differs notably. Accordingly, some literature distinguishes between the common infantile form, and the atypical congenital form and juvenile form [75, 83]. Unlike the infantile form, where symptoms appear after around 3 months, in the congenital form, they appear around or a few days after birth, often leading to death within several days or weeks. Contrary, symptoms in the juvenile form are delayed with the initial symptoms appearing later in life [83]. There are many examples of juvenile CD patients [93–103]. Notably, in certain cases, some symptoms (delayed motoric milestones) appeared early, as seen in classical CD. However, the disease progression remained milder than usual, indicating that even milder and juvenile forms may manifest and be noticeable at the early stages [102, 104]. Conversely, one study reports that six patients with infantile-onset CD survived beyond six years of age, but points out that this might be the result of better medical management and care, rather than evidence of genetic heterogeneity [82]. Indeed, a later paper concludes that the clinical course of CD patients was not due to mutation heterogeneity, but rather reflects the improvement in patient care and other unrelated factors [105]. The observation is corroborated by another study noting that “*prolonged survival of patients with early-onset disease, even into the second and third decade, is not uncommon*” [78].

Interestingly, one report found no obvious correlation between the severity of the WM degeneration and the clinical presentation [106]. Likewise, another study noted that neither seizures nor basic psychomotor skills (visual tracking and head control) within the first two years of life had a statistically significant influence upon survival [74]. Hence, specific CD symptoms may not always reflect the severity of the disease but vary from patient to patient.

Some literature on variability in the manifestations of the disease, did not find evidence supporting the three distinct forms of CD [78]. Likewise, the categorization was described as “flawed” by a later study, which instead argued for using gene-based diagnosis of typical versus mild Canavan disease [24]. Consequently, rather than three distinct forms, CD phenotypes are likely better described as a spectrum of severity, with the possibility of specific symptoms being more or less pronounced due to genetic or environmental factors. More recently, a CD severity score with assessment of 11 symptoms and abilities was developed [74], which may help to systematically and objectively evaluate CD, to compare across studies, and potentially assign severity to specific *ASPA* alleles.

Possibly the CD severity, at least in part, reflects the patient genotype, with ASPA variants with slight residual activity resulting in milder forms of CD. However, other genetic or environmental factors may play a role as well [40, 78]. Thus, in a juvenile CD case with two sisters both heterozygous with the same alleles (A305E and R71H), they presented with developmental delays from 19 and 50 months, respectively, suggesting other factors than genotype play a role as well [96].

Another study reported that two CD patients homozygous for A305E variant had a milder phenotype, although most CD patients homozygous for the variant had early onset of CD and severe symptoms [93]. Likewise, in one study of 23 CD patients, macrocephaly seems to occur slightly earlier in girls (7 months) than in boys (8.5 months), hinting towards sexual differences in manifestation of that specific symptom [74].

Phenotypic variation is also seen in the *Aspa*^−/−^ mice, where some died shortly after weaning, while others survived between 1.5 and 9 months [28]. Differences in severity are also evident between the different *Aspa* knock-out mouse models [107] (Box 1).

So far, attempts at linking specific variants to CD severity have been limited [43, 63, 64, 74, 93, 101, 108]. However, variants likely to be mild may include: G274R, P181T, Y231C, P257R, I143T, K213E, R71H, Y288C, I170T, G101V and D204H [63, 64, 101]. Given the recessive nature of the disease, variant effects on CD severity needs to be considered in context of the variant expressed from the other *ASPA* allele. A mild variant may not cause CD at all in a homozygous setting but could yield a mild phenotype when combined with a detrimental variant. Given the ASPA homodimer formation, specific variant combinations may also potentially result in positive or negative epistasis, adding further complexity to the matter.

### Box 1 Animal models for Canavan disease

An *Aspa* knock-out mouse model was constructed by homologous recombination of an *Aspa* construct with a 10 bp deletion in exon four in ES cells, which were subsequently injected into C57BL/6-*Tyr*^*c−Brd*^ blastocysts [28]. In accordance with the recessive nature of CD in humans, heterozygous CD mice had no overt phenotype, whereas homozygous mice displayed various CD symptoms [28]. For instance, the *Aspa* homozygous knock-out mice, had lower weight (7.23 ± 0.93 g at weaning) compared to heterozygous or wild-type littermates, as well as age and gender-matched controls (14.06 ± 0.84 g) [28]. The CD mice also had a reduced lifespan, with a few mice dying shortly after weaning and others surviving for between 1.5 and 9 months. Other symptoms include macroencephaly with craniofacially abnormalities, ataxia (tremors, splayed legs, slower shaky pace, reduced mobility) with a reduced performance on rotarod tests (1.16 ± 1.69 s vs. 44.9 ± 21.4 s for wild-type and heterozygous controls). The mice were also lethargic, and a subset developed seizures at 6 months. Urine NAA levels were approximately eightfold higher in CD mice (1,541 ng/mg creatinine) vs heterozygotes (184 ng/mg creatinine) or WT (170 ng/mg creatinine). Lastly, brain scanning revealed abnormalities resembling CD.

Two other CD mouse models have since been created. In 2003 [337], the mutagen N-ethyl-N-nitrosourea was used to create a nonsense mutation Q193X, in the *Aspa* gene. This strain is known as *Aspa*^Nur7^ [337] and later characterized as a new CD model [86]. In 2011, an aspartoacylase-lacZ knock-in mouse model was engineered, where the bacterial β-galactosidase (*lacZ*) gene is inserted after the *Aspa* regulatory elements to abolish *Aspa* expression, [30]. This strain was later used to demonstrate altered central auditory processing, with impaired speed of nerve conduction and hypomyelination of in the central auditory system [40].

In addition to the mouse models, a naturally occurring rat CD model, known as the tremor rat, has been utilized for in vivo studies. The tremor rat carries a deletion spanning at least 200 kb including the four genes, encoding an olfactory receptor, ASPA, the vanilloid receptor subtype I, and the Ca^2+^/calmodulin-dependent protein kinase IV [27].

While all models show resemblance to CD patients, Ahmed et al. [107] point out that compared to the original mouse model from 2000, the three other rodent models have less severe phenotypes and near normal lifespans. Likewise, Mersmann et al. [30], point out that neurological phenotypes varies considerably between different rodent models.

When working with the tremor rat model, it is hard to attribute phenotypes specifically to ASPA-deficiency rather than other effects of the deletion [15]. Since the rat brain arguably resembles the human brain more than the mouse brain, a clean ASPA deficient rat model would be desirable.

### NAA metabolism and the normal role of ASPA

**Fig. 5:**
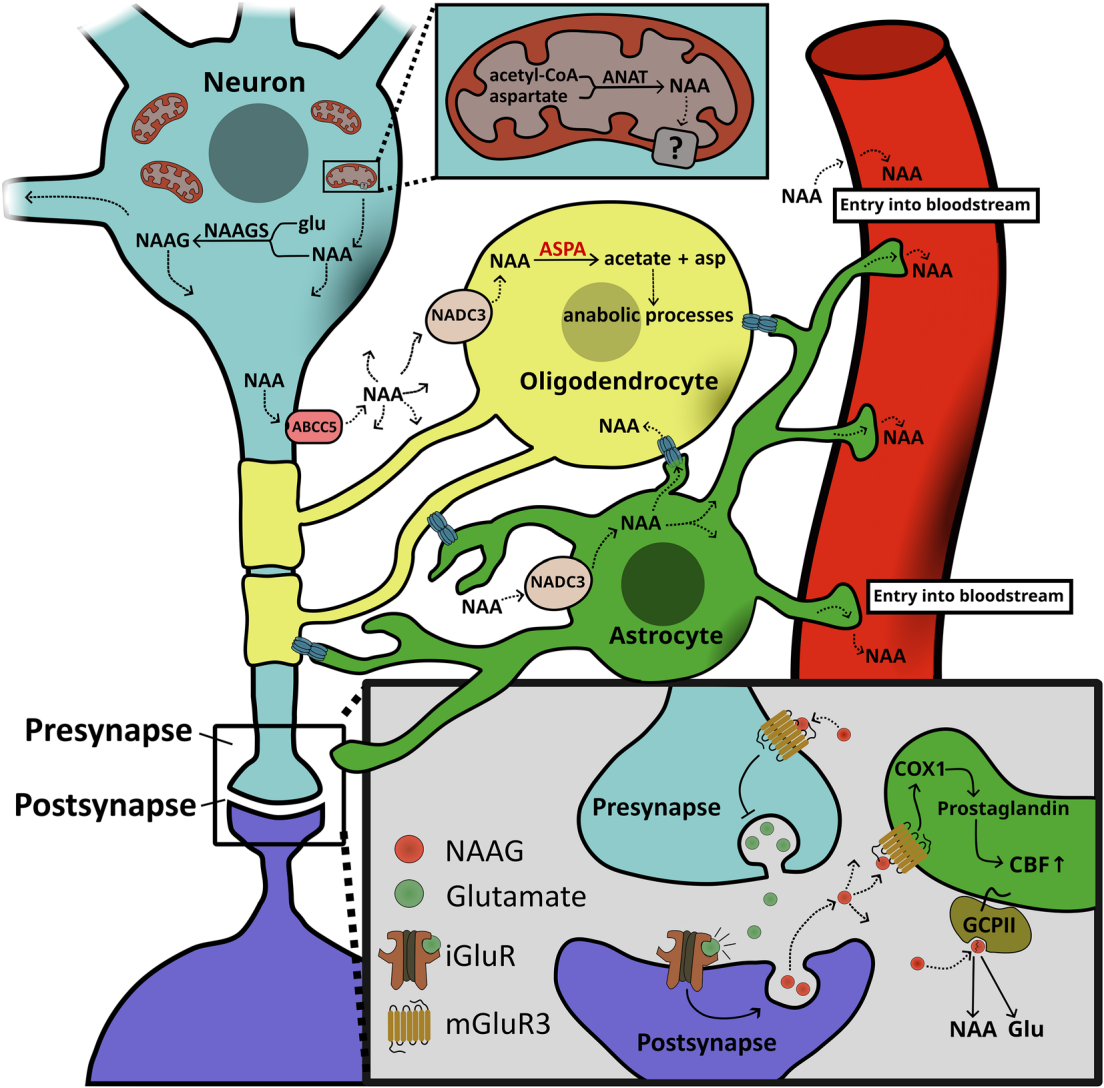
*Overview of the NAA cycle.* NAA is synthetized from acetyl-CoA and aspartate in the mitochondria of neurons by the enzyme aspartate N-acetyltransferase (ANAT), and subsequently transported to the cytosol by unknown transporters. NAA might either be released from neurons or converted into N-acetyl-aspartyl glutamate (NAAG) catalyzed by NAAG synthetase I or II (NAAGS). NAA release likely occurs through ABCC5 as well as other uncharacterized transporters. Upon its release, NAA may be taken up by astrocytes or oligodendrocytes through the sodium-dependent dicarboxylate cotransporter 3 (NADC3) or exchanged through gap junctions. Within oligodendrocytes, ASPA hydrolyzes NAA to aspartate and acetate, which can be utilized by the cell. A fraction of NAA may also end up in the bloodstream. NAAG can be released from postsynaptic dendrites in response to stimulation of ionotropic glutamate receptors. NAAG then acts on presynaptic metabotropic glutamate receptor 3 (mGluR3) to inhibit further presynaptic glutamate release. Additionally, NAAG acts on mGluR3 on astrocytes to induce cyclooxygenase (COX1) activation, which in turn leads to release of prostaglandins to the vascular system. This in turn increased cerebral blood flow (CBF) to the area. Lastly, glutamate carboxypeptidase II/III (GCPII/III), catalyse the hydrolysis of NAAG to NAA and glutamate

The ASPA substrate, NAA, is found in high concentrations within the brains of mammals and birds [109–112]. Within the human brain, it is found in concentrations of ~ 10 mM depending on the specific brain area [113, 114], thus making it one of the most abundant amino acids in the brain, second only to glutamate [56]. NAA is synthetized within the mitochondria of neurons [115–117] from aspartate and acetyl-coenzyme A by the enzyme aspartate N-acetyltransferase (Asp-NAT or ANAT) [118, 119] encoded by the *NAT8L* gene [120] (Fig. 5).

Through an unknown transportation mechanism from the mitochondria to the cytosol, NAA accumulates in the neurons, reaching concentrations of up to 20 mM and accounting for 7% of neuron osmolarity [11]. From here NAA is excreted from the neurons. While the efflux of NAA from neurons is poorly understood, it is likely driven by the high intracellular/extracellular NAA concentration gradient [121], likely along with water molecules, thus serving as a means for neurons to expel metabolic water [12, 114, 122]. Transport has been speculated to occur via members of the solute carriers (SLC) superfamily [123]. Most likely, at least some of the efflux can be ascribed to the ubiquitous efflux transporter ABCC5, which is part of the superfamily of ATP-binding cassette (ABC) transporters [124, 125].

#### NAA release mechanisms

Some data suggest that NAA and its derivative N-acetyl-aspartyl glutamate (NAAG) (further discussed below) are released in response to neuronal depolarization, although there seems to be conflicting data as to whether the release is Ca^2+^-dependent.

One study found that transient (5 min) N-methyl-D-aspartate (NMDA)-receptor activation (60 µM) induced a long lasting, Ca^2+^-dependent efflux of NAA in preparations of organotypic slices of rat hippocampus. Interestingly, the NAA efflux did not seem to be directed to cell swelling or depolarization, but rather coupled to Ca^2+^-influx via the NMDA-receptor. The efflux also seemed to persist for at least 20 min after the omission of NMDA [126].

One rat brain slice superfusion study found that a basal efflux of NAA and its derivate NAAG occurred, which could be increased by 300% by electrical stimulus, that was sensitive to a voltage-dependent Na^+^ channel blocker, tetrodotoxin [127]. Likewise an older study used the same technique to demonstrate a largely Ca^2+^-dependent increase in released NAAG in superfusates from rat neocortex, piriform cortex/amygdala, and hippocampus upon depolarization with 50 mM K^+^ [128]. In rat microdialysis studies examining K^+^-induced local depolarizing stimuli, NAA levels in the extracellular fluid (ECF) were shown to consistently increase after the stimuli [121, 129], with one of the studies showing that the release occurred irrespective of whether or not Ca^2+^ was present in the perfusion medium [121].

In magnetic resonance spectroscopy (MRS) studies of NAA, it was shown that photo-stimulation leads to a decrease in NAA levels, likely corresponding to released and subsequently degraded NAA [130]. Later, the same drop in NAA level was shown in rat prefrontal cortex [131] and again in humans [132]. It should be noted, however, that a newer study found that NAAG and NAA in the visual cortex remained constant during continuous visual stimulation [133]. Thus, while we know NAA/NAAG is released, the mechanisms behind the release remain poorly understood. It has been suggested that this efflux of NAA, may be a mechanism of osmoregulation [12, 121, 130, 134, 135].

#### NAA catabolism and the role of ASPA

Upon its release from neurons, NAA has been suggested to follow a major and minor pathway [11]. In the major pathway, NAA is absorbed by oligodendrocytes and hydrolyzed by ASPA into acetate and aspartate, which, in turn, can be utilized by oligodendrocytes, astrocytes, neurons or passed via the ECF into systemic circulation for use in other tissues. In the minor pathway, NAA may travel from the cerebrospinal fluid (CSF) into the bloodstream and subsequently utilized by other cells or excreted in the urine in barely-traceable quantities [11, 136] (Fig. 5).

The average lifetime of NAA has been estimated to 16–18 h in healthy individuals [12, 114, 137]. In CD patients, the lifetime is likely longer at around 24–48 [11] or 62 [12] hours. In addition, the synthesis rate of NAA in human brain in vivo was measured to 9.2 ± 3.9 nmol/min per g in controls and 3.6 ± 0.1 nmol/min per g in CD patients [137], suggesting a negative feedback regulation of NAA on its synthesis.

#### NAA uptake and transport mechanisms

Like its excretion from neurons, the uptake of NAA into glia cells is not well described. The sodium-dependent dicarboxylate cotransporter 3 (NaDC3) encoded by the *SLC13A3* gene has been shown to be responsible for NAA uptake, along with three sodium ions, in rat astrocytes [138, 139]. Since the transporter is also found in oligodendrocytes [123, 140, 141], NaDC3 is a potential candidate for NAA transport into oligodendrocytes and, to our knowledge, the only transporter suggested in the literature. Accordingly, studies have shown increased NAA levels in the urine of *Slc13a3* homozygous knock-out mice [142]. Although this further emphasizes the importance of NaDC3 in NAA uptake [123], it is, without the use of tissue-specific knock-out strains, not possible to conclude whether this effect is due to the lack of NaDC3 in the brain or in the kidneys, where NaDC3 is also found within the proximal kidney tubule cells [143].

Alternatively, connexins (combined in hexameric complexes known as connexons) form gap junctions between astrocytes and oligodendrocytes, thus forming a “glial syncytium”, which may allow the transport of NAA from astrocytes to oligodendrocytes [123, 144].

Rather than connecting with a connexon on another cell, connexons may also form hemichannels, which enable the release of metabolites to the extracellular space. Many gap junctions are found between astrocytes (astrocyte-astrocyte junctions) while there are fewer astrocyte-oligodendrocyte junctions, and few or none between oligodendrocytes themselves or neurons and glia cells [144].

The astrocyte-oligodendrocyte (A/O) gap junctions have been found between astrocyte processes and the oligodendrocyte cell body, its processes, and its outer (abaxonal) layer of the myelin sheath in both WM and GM [144]. The astrocyte-oligodendrocyte gap junctions include connexins Cx26, Cx30, and Cx43 expressed in astrocytes and Cx29, Cx32 and Cx47 expressed in oligodendrocytes [144, 145]. Intriguingly, the importance of connexins in CD seems supported by detrimental effects on oligodendrocytes and myelination observed upon loss of some A/O connexins in both humans and in mice models [146–150]. As has been pointed out by multiple authors [113, 123], these phenotypes resemble those of CD. However, they do not necessarily relate to NAA transport. For example, gene variants in Cx32 have been linked to X-linked Charcot–Marie–Tooth disease, where demyelination occurs in the peripheral nervous system rather than the CNS. Considering that the role of NAA mainly involves the CNS, this suggests that connexins may cause demyelination through mechanisms independent of NAA transport [146]. Indeed, it is easily imaginable that many other implications of defective connexins on astrocytes and oligodendrocytes can disrupt processes such as myelination. However, so far, no studies have addressed NAA transportation in the context of defective connexins and their potential role in the disease phenotype.

#### N-acetyl-aspartyl glutamate

NAA is generally not considered a neurotransmitter, although one paper argues otherwise, suggesting it acts on the G protein-coupled metabotropic glutamate receptor (mGluR) to induce an inward current that results in excitation of the neurons [151]. However, its derivative N-acetyl-aspartyl glutamate (NAAG) fulfills most criteria of a neurotransmitter while likely also being widely distributed and the third most prevalent transmitter in the mammalian nervous system after glutamate and γ-aminobutyric acid (GABA) [152, 153] as well as the most abundant dipeptide in the brain [154]. Interestingly, the brain distribution of NAA and NAAG seems to be distinct [155].

NAAG is synthesized from NAA and glutamate by NAAG synthetase I and II in neurons [156, 157]. Upon stimulation of ionotropic glutamate receptors on the dendrites of postsynaptic neurons, NAAG is secreted and acts as an agonist on the presynaptic metabotropic glutamate receptor 3 (mGluR3), thereby reducing further glutamate release [157–159]. In addition to this retrograde NAA release, presynaptic NAAG release has been reported in retinal neurons [160] and in the neuromuscular junction [161].

NAAG also acts on mGluR3 on astrocytes [125, 159] to induce activation of the cyclooxygenase COX1 in astrocytes leading to secondary release of prostaglandins to the vascular system. This in turn induces a hyperemic response leading to increased blood flow *i.e*. (increased oxygen and glucose) to the area [125]. Consequently, NAAG plays a role in regulating cerebral blood flow [125, 162]. Equally important, NAAG is catabolized to glutamate and NAA by the membrane anchored carboxypeptidase II [163] and III [164] (GCP-II & GCP-III) expressed on the extracellular face of astrocytes [113, 163–166], thus constituting a second source of NAA (Fig. 5). Lastly, it has been reported that NAAG is further modified to N-acetylaspartyl-glutamylglutamate (NAAG2) by NAAGS-II, although the significance of this product is poorly described in the literature [156].

#### NAA and NAAG release in grey and white matter

One aspect likely useful for elucidating the role of NAA and its related metabolite NAAG in relation to CD, is the distinction between release of the compounds in the brain WM and GM, respectively. However, not much literature has focused on this.

In healthy individuals, it has been reported that NAA levels are higher in GM than in WM, likely due to the greater neuronal density in GM [167–169]. Contrary, NAAG is found in higher levels in WM than GM [170–172]. In line with these observations, Baslow and Guilfoyle [13] argue that NAA efflux from neurons to ECF occurs in GM in response to neuronal stimulus as a means of fulfilling a osmoregulatory role of NAA, whereas in WM the only source of NAA is from the catabolism of NAAG. The authors point out that GM is highly vascularized, the primary site for energy production, and includes neurons with unrestricted surfaces, which allows afflux and NAA/water efflux to occur readily, unlike WM where such exchange is highly limited due to axonal myelination [13]. They also point to the high NAAG peptidase activity in WM [166], and the fact that of all known metabotropic glutamate receptors, only the target receptor for NAAG, named GRM3, is present in WM [13, 173].

#### NAA and NMR

Due to its abundance and high visibility with nuclear magnetic resonance, NAA has proven useful for MRS and magnetic resonance imaging (MRI) in the brain [71, 174, 175]. The three equivalent hydrogen atoms of the acetate group resonate in NMR with a single, sharp peak, with a chemical shift of 2.02 ppm relative to the standard tetramethylsilane. While NAA is responsible for the majority of the signal, NAAG, *N*-acetylneuraminic acid, and underlying coupled resonances of glutamate and glutamine also contribute. In particular NAAG may contribute 15% to 25% of the peak signal [113].

NAA has also been considered a marker of neuronal health, with low NAA levels, indicative of poor neuronal health, being observed in multiple neurological diseases such as schizophrenia, amyotrophic lateral sclerosis, multiple sclerosis, epilepsy, Alzheimer’s disease, and Parkinson’s disease [49, 71, 88, 175]. Contrary, an elevated NAA level is an indication of CD [113].

#### Other roles of NAA

While NAA is clearly most well-described for its roles in the brain, it also seems to play a role in several other cell types. Indeed, *NAT8L* is also expressed in brown adipose tissue (BAT) at levels comparable to the brain, and to a lesser extent in white adipose tissue (WAT) as well, suggesting NAA synthesis also occurs in these cell types [176]. Notably, *ASPA* is also expressed in both BAT and WAT at levels similar to the brain [177], and a role of NAA in these tissues seems further supported by the phenotype of ASPA-deficient mice (lower total body-fat percentage and weight) [41, 178]. Interestingly, body weight is restored to wild-type levels in *Nat8L/Aspa* double knock-out mice [41]. It should be noted that Madhavarao et al. [179] reported no difference in animal weight between WT and ASPA KO mice. However, this is likely due to the age of the animals, as 17 days old animals were, while in Surendran et al*.* the weight difference is only noticeable with four week old mice [178] and in Jonquieres et al. [41], the weight difference was not evident between two and six months.

It has also been suggested that NAA, through the minor pathway, may provide a brain-specific mechanism for excretion of aspartate-associated nitrogen, similar to the synthesis of urea in the liver [113]. Supporting this idea, is the readiness by which NAA is transported out of the brain, which becomes apparent in CD patients where the NAA levels rise much more in the urine than in the brain [113, 180]. Removing aspartate from neurons through acetylation of NAA would also favor α-ketoglutarate formation from glutamate (catalyzed by aspartate aminotransferase), thus improving energy production through the TCA cycle. This would help accommodate the large energy demands of neurons, without producing ammonia, as would occur when α-ketoglutarate is produced via the glutamate dehydrogenase reaction. Considering these roles of NAA in neuronal energy metabolism, its aforementioned use as a marker of neuronal health is hardly surprising [88].

Furthermore, it has been suggested that NAA may serve as a reservoir for glutamate, thus negating the cytotoxic effects of high glutamate levels [181, 182]. Indeed, NAA and glutamate are inherently linked through metabolic pathways, most notable the tricarboxylic acid and the glutamate–glutamine cycles [113]. A similar role as glutamate reservoir could be proposed for NAAG as well [183].

Between the high NAA abundance in the brain, conserved only within the animal classes with the most advanced brains (birds and mammals) [109], its fast metabolic cycle across multiple cellular compartments, elusive transport mechanisms within the body and importance for neurotransmitters such as NAAG and glutamate, it is hardly surprising that NAA has been mentioned as the most enigmatic free amino acid in the human brain [11].

### NAA in Canavan disease

In CD patients, NAA hydrolysis in the oligodendrocytes is abolished. This confers major changes to NAA and associated metabolites. As NAA cannot be hydrolyzed by oligodendrocytes in the brain, more NAA is removed through the minor pathway, where NAA passes from the ECF to the bloodstream and is eventually filtered out to the kidneys. This process leads to the elevated NAA levels in blood (NAA acidemia) [12, 88] and urine (N-acetylaspartic aciduria) [11, 32, 88, 114, 184].

In CD, the neuronal NAA synthesis persists, although only at approximately 38% of the normal rate [12, 114, 137]. Notably metabolic consequences of deficient ASPA activity within the brain also includes loss of the high neuronal NAA-gradient, which may reduce the efficiency of NAA export from these cells. Additionally, as the aspartate from NAA cannot be reused for NAA-synthesis, there is an aspartate deficit, which must be made up for by aspartate production from other metabolic resources [184].

Although ASPA is widely considered as the only known enzyme that can metabolize NAA [185], the enzyme amidohydrolase I, which is highly expressed within astrocytes in the brain and kidney [186, 187], might be able to hydrolyze NAA to some extent, based on results from protein purified from rat [47] and trout [188] brain. This contribution may play a role in CD patients, where ASPA functionality is abolished, but obviously does not restore oligodendrocyte-specific hydrolysis [114].

NAA levels in the brain of CD patients were found to be elevated by approximately 50% compared to healthy controls [11, 24, 189], although one study reported NAA to not be significantly elevated [190]. Based on older publications, the normal NAA brain WM level is 5–10 mM as opposed to 15–20 mM in untreated CD patients [191].

In healthy individuals, NAA levels in urine have been reported in the range from 5 to 20 mmol/mol creatinine, whereas the levels in CD patients were in the range of 391–3073 mmol/mol creatinine [69, 192]. Notably, NAAG was also elevated in the urine of CD patients [193]. In the CSF of a CD patient, the NAA concentration was measured at 611 μmol/L, whereas in 10 control samples, the level was below the detection limit of 2.3 μmol/L [69]. In the same CD patient, the blood NAA concentration was 7 μmol/L [69], as opposed to 0.11 mmol/L [194] and 0.44 μmol/L [195] in healthy individuals. This low concentration compared to urine levels suggests NAA is effectively filtered out of the blood in CD patients [69]. In addition to the elevated blood NAA levels, the NAA aciduria may also be partly explained by the inability to recycle NAA in the kidneys [113], since ASPA activity in kidneys will also be non-functional in CD patients. Notably, variations in measuring techniques and fluctuations between CD patients mean that NAA values should be viewed as indicative rather than absolute defined values.

In addition to elevated NAA levels, 46% reduced glutamate levels were also observed in a CD patient [71, 189]. In the brain of *Aspa* homozygous knock-out mice, glutamate and its metabolic product γ-aminobutyric acid (GABA), were also shown to be reduced [71, 178, 196], while aspartate was elevated [178]. The reduced glutamate levels, and elevated aspartate levels observed in mice, was by the same study proposed to be related to the reduced aspartate aminotransferase activity reported in the CD mouse model [178]. But whether this is the cause or effect of a dysfunctional metabolism caused by lack of aspartoacylase is hard to say. A differential gene expression analysis of a CD patient found that glutamate, and aspartate metabolism, were significantly dysregulated, along with changes in genes involved in apoptosis, muscle contraction and development, mitochondrial oxidation and inflammation [197]. Indeed, it is possible that indirect metabolic dysfunctions from lack of NAA catalysis plays a significant role in causing or aggravating the symptoms of Canavan disease [184], a notion supported by the benefits of dietary acetate supplementation (as discussed further in the following section).

### Etiology of Canavan disease

Various hypotheses have been proposed to explain the etiology of Canavan disease. Notably, several of these are not mutually exclusive, and therefore likely need to be considered in combination to explain the full pathology of CD. In the following, we will summarize the main findings that have led to the present understanding of CD etiology.

#### Neuronal toxicity

One possibility is that NAA accumulation leads to neuronal excitotoxicity [123], similar to what has been reported for glutamate [198]. It was shown that direct injections of NAA (4 or 8 µmol doses) into normal Wistar rats, led to seizure-like symptoms resembling those observed in CD patients, and lower doses (2 µmol) were sufficient to induce the same seizures in tremor rats [27, 199]. However, presuming NAA is not a neurotransmitter, direct neuronal excitotoxicity of NAA seems unlikely. Rather the seizures observed in CD patients, may be caused by resulting misbalances from the disrupted NAA metabolism such as reduced glutamate (and GABA based on mice studies) [189] and potentially higher NAAG-levels [193, 200], both of which are reported neurotransmitters [152, 201, 202]. A general cytotoxic effect of NAA also seems unlikely, as NAA appears to be elevated in GM [167–169], while CD mainly affects the WM. In addition, feeding Sprague–Dawley rats NAA at 500 mg/kg of body weight/day, administered for two consecutive generations, showed no changes in neurobehavioral tests [203]. This could, however, have been due to lack of neuronal uptake, breakdown in the gut or catalysis by ASPA. However, in another study, feeding healthy mice N-acetyl-aspartate monomethyl ester until their brain NAA-levels were comparable to those of CD mice, did not elicit neuropathological abnormalities [204]. In more recent data based on three-dimensional human iPSC-derived myelin spheroids consisting of neurons, astrocytes and myelin sheath-forming oligodendrocytes, adding 5 mM NAA had a toxic effect on oligodendrocyte myelination [205].

#### The osmotic-hydrostatic hypothesis

The “osmotic-hydrostatic/molecular water pump” hypothesis goes all the way back to Canavan’s case report, in which moderate to extreme edema and signs of increased cerebral pressure were reported [65, 114]. According to this theory, the cycling of NAA from neurons to oligodendrocytes normally acts as a molecular water pump facilitating the transport of water out of neurons [19, 122, 206]. This is accomplished by utilizing the high intracellular/extracellular NAA gradient of neurons to drive water transport up its gradient [12, 19, 206, 207], similarly to what is seen in the efflux N-acetyl-L-histidine water pump [208] and the influx Na^+^-glucose cotransporter [209, 210]. In CD patients, loss of the osmoregulatory role of NAA and its accumulation could explain the microencephaly observed in CD patients, as well as the widespread leukodystrophy [11, 13]. Likewise, loss of NAA catalysis in myelinating oligodendrocytes, would lead to an accumulation of NAA in the in periaxonal space, increasing the osmotic pressure and subsequently causing the intramyelinic splitting, interlamellar edema and breakage at the paranodal seals, which constitutes the dysmyelination characteristic of CD. This could also explain the vacuolization that is observed in the deep layers of cortex and subcortical WM in progressed stages of CD [11, 122], as well as the macrocephaly [74], increased CSF pressure [122, 211] and indications of increased water content in the brain [24].

Indeed, NAA is an important osmolyte in the brain, estimated to constituting 1% of the brain's dry weight and 3–4% of its total osmolarity [11], likely making it capable of such an osmoregulatory role. Additionally, NAA efflux is thought to occur along with at least 32 water molecules and a cation [114, 134]. The estimated NAA lifetime of ~ 17 h in healthy individuals, compared to > 24 h in CD patients, would also support the notable reduction in the osmoregulatory roles of NAA in CD patients, lending further credibility to the hypothesis [11, 137]. Speaking against the model, however, is the fact that elevating NAA to supraphysiological levels by overexpressing *Nat8L* did not elicit any neurological deficits [41].

#### The acetyl-lipid myelin hypothesis

Another theory is the “acetyl-lipid myelin/oligodendroglial starvation” hypothesis, originally proposed in 1966 [212]. This hypothesis states that the acetate released from NAA-hydrolyzation in the oligodendrocytes is important for the lipid synthesis required to make the myelin sheaths. Consequently, in CD patients, oligodendrocytes are unable to acquire sufficient acetate from NAA hydrolysis, leading to improper myelin sheath development, thus explaining the observed dysmyelination [8, 88, 114, 212].

Supporting this theory is the well-documented incorporation of NAA-derived acetate into myelin lipids [114, 212–216]. Furthermore, in rats and other mammals, the rapid rise in NAA levels [109, 110, 214, 217] and *ASPA* expression [8, 37, 39] in the first few postnatal weeks, coincide with a period of high myelination [218, 219]. Since myelination also occurs prenatally in humans [219], as opposed to mainly postnatally in rats [218], it would be interesting to investigate if this is accompanied by an earlier rise in NAA and aspartate levels as well.

Speaking against this model, abolishing NAA synthesis by deletion of the acetyl aspartate synthase gene (*Nat8L*) in mice does not seem to affect myelination [220, 221]. The lack of NAA would be expected to cause severe dysmyelination, assuming the acetyl-lipid-myelin hypothesis holds true. However, in a patient with no visible NAA or NAAG spectra (*i.e.* likely deficient for *Nat8L*, though not verified genetically) a retardation phenotype, similar to Canavan disease and moderately delayed myelination was reported [222–224], contradicting the mice studies.

Another study in CD mice, found that the abolishment of *Nat8L* suppressed the loss of cerebral cortical and cerebellar neurons otherwise seen in these mice [225], perhaps supporting the osmotic-hydrostatic hypothesis.

Also going against the acetyl-lipid myelin theory is the fact, that restoring ASPA activity in astrocytes rather than oligodendrocytes seems to be sufficient to prevent the CD phenotype [226], although these results could be explained by transfer of NAA-derived acetate or similar metabolites from astrocytes to oligodendrocytes.

The acetyl-lipid myelin-hypothesis also fails to account for the cellular and extracellular edemas observed in CD. Likewise, opponents point out that acetate could be provided to oligodendrocytes by other means than NAA from neurons, e.g. via glucose from astrocytes, indicating that the NAA has additional purposes than merely being a carbon source [13, 114].

#### The oxidative stress hypothesis

In one study, tremor rats, which were continuously fed glyceryl triacetate (GTA) from 1 week after birth, showed improved motor performance and myelin galactocerebroside content as well as modestly reduced vacuolation [227]. This on one hand suggests NAA is not necessary for myelin sheath development, but simultaneously points to acetate-deficiency as an underlying cause of at least some of the Canavan disease symptoms.

Following that line of thought, the third “oxidative stress” hypothesis, proposed by Francis et al., points at increased oxidative stress due to the deficient NAA-catabolism as the underlying cause of Canavan disease. Supporting this hypothesis are findings that show oxidative stress occurring before oligodendrocyte dysmyelination in the homozygous *Aspa*^*Nur7*^ mouse model [228]. Later, the same group demonstrated how dietary triheptanoin (a synthetic triglyceride with a carbon chain length of seven, which can be catabolized to provide as a source of acetate [15, 123]) administration reduced oxidative stress and alleviated CD symptoms by increasing myelination, reducing spongyform degeneration and improving motor function. Importantly, this treatment was effective only in younger mice, highlighting the significance of early postnatal myelination events and establishing the therapeutic intervention window accordingly [15]. The theory gains further support from observations showing reduced levels of acetyl-CoA and ATP in ASPA-deficient mice [87, 179, 229], and the fact that NAA is essential for juvenile *Nat8L* knock-out mice, when on a fat-free diet [120]. Another study found that feeding mice with glyceryl triacetate, which provided a significantly better acetate source than calcium acetate, showed no overt pathology, and increased acetate levels, but not NAA levels [230]. Likely supporting both the oxidative stress and acetyl-lipid myelin-hypothesis, knock-down of ASPA in immortalized brown adipocytes indicated deficiencies in acetyl-CoA and lipid metabolism as shown by transcriptome analysis [177].

#### Dysregulation of the malate aspartate shuttle

Some of the CD phenotypes may also be the result of dysregulation of the malate aspartate shuttle (MAS) [231]. Mainly important in neuronal cells, MAS is considered to be the major redox shuttle system responsible for maintaining the NAD^+^/NADH ratio at levels favorable for the oxidative metabolism of glucose [232]. Considering *Aspa* knock-out mice have been reported to have elevated aspartate levels and reduced glutamate levels [178], both of which are related to MAS, one might suspect some of the phenotypes of CD patients to be caused indirectly by dysregulation of MAS. Interestingly, deletion of the MAS gene *Aralar* (AGC1/SLC25A12) in mice leads to a drop in aspartate and NAA levels, highlighting its involvement in the NAA cycle [233]. These mice also exhibit hypomyelination, and reduced levels of myelin-specific brain lipids, particularly galactocerebrosides, resembling what is seen in CD [232–235]. In one observed human case of *ARALAR* homozygous for the Q590R missense variant, symptoms also conspicuously resembled CD, with normal development during the first months of life, followed by delayed psychomotor development and seizures at months 5 and 7, respectively, poor head control, severe muscular hypotonia, psychomotor retardation and global hypomyelination [236].

#### Dysregulation of histone acetylation and oligodendrocyte differentiation

Finally, is has been proposed that lack of available acetate may affect acetylation of histones which in turn, is important for oligodendrocyte differentiation [123]. Studies of oligodendrocyte maturation have highlighted the important role of histone acetylation and deacetylation in the epigenetic control of cellular differentiation from oligodendrocyte precursor cells to mature oligodendrocytes [237, 238]. Similarly, highly acetylated nuclear histones H2B and H3, indicative of the existence of non-compact chromatin as seen during early development, were detected in the WM of adult ASPA knock-out mice [239]. Another study performed on oligodendrocyte cultures also showed that NAA treatment resulted in alterations in the levels of histone H3 methylation, including H3K4me3, H3K9me2, and H3K9me3 [240]. While intriguing, a more concise cause-effect relationship needs to be established to corroborate this hypothesis.

More recently, a study in mice showed loss of ASPA activity to shift oligodendrocyte and neuronal markers towards a less differentiated state, which could be improved or normalized by reconstitution of ASPA activity [241]. However, further elucidation of the connection between ASPA activity and cell differentiation is needed if this data is to favor one hypothesis over another. Indeed, as oligodendrocyte myelin sheath synthesis, energy metabolism and epigenetic modifications are inevitably interconnected, the acetyl-lipid myelin-hypothesis, oxidative stress hypothesis and histone acetylation hypothesis may perhaps favorably be lumped together as one larger hypothesis, depending on the results of future studies.

Evidently, further studies into the NAA cycle and its cellular compartments may provide valuable information on the etiology of CD. For instance, using a conditional knock-out to induce ASPA deficiency after the myelination has occurred may reveal the relative contribution from the acetyl-lipid myelin hypothesis and the osmotic-hydrostatic hypothesis, since the prior seems to be mostly relevant for the early stages of life, where myelination is extensive, whereas one would expect the latter theory to be detrimental even after the myelin sheaths have developed. In addition, standardized tests for assessments of CD symptoms in rodent models would help in comparing the results from individual studies.

### Possible treatments

Much effort has been put into means to ameliorate the symptoms of Canavan disease and to explore the possibilities for a cure. Examples of palliative treatment include provision of proper nutrition and hydration [19] as well as treatment of seizures with anticonvulsants [19, 75] including acetazolamide, clonazepam, oxcarbazepine, phenobarbital and valproic acid [24]. In an unusual case of a CD patient, clobazam and primidone were administered to prevent frequently intractable seizures, after previous anticonvulsant treatment using phenytoin, levetiracetam and phenobarbital proved unsuccessful in getting the seizures under control [242].

Canavan patients may often benefit from machines assisting in respiratory functions, nebulizers to help administer medication, diverse positioning equipment to help accommodate the hypotonia and feeding pumps [75, 211].

In line with the main hypothesis for CD etiology, most drug treatments have aimed at reducing brain NAA levels and intracranial pressure or provide a supplement to compensate for lost NAA catabolism. Following the success of glyceryl triacetate (GTA) in *Aspa* knock-out rat [227, 243] and mice [230] models [227, 230], the treatment was tested on humans. While GTA-treatment was well-tolerated, no improvements in motor function were observed, possibly due to the age of the patients (8 months and 1 year [244] & 8 and 13 months [243], respectively). Accordingly, Segel et al. [244] point out that an earlier intervention time point within the first to 3 months of life and before severe symptoms—indicative of irreversible brain damage—appear, is likely to yield better results. In addition, increasing the GTA dosage might be considered given the lack of adverse effects in studies with the compound [227, 230, 243, 244]. By the same rationale, triheptanoin could potentially have beneficial effects as well [15, 123].

Acetazolamide, an carbonic anhydrase inhibitor with diuretic properties [245] and also an anti-seizure drug [246], was demonstrated to reduce the intercranial pressure in CD patients, but not the water content or NAA levels [19, 67, 211, 247].

Lithium and sodium valproate were shown to reduce NAA levels in rats [248]. Likewise, ethanol, pyrazole and several pyrazole-derivatives also demonstrated an ability to reduce brain NAA concentrations [249]. However, when tested in tremor rats, only lithium chloride and not ethanol, pyrazole-compounds nor valproate were able to lower brain NAA levels [250]. Inspired by these findings, lithium citrate has been tested on humans on multiple occasions, with no signs of toxicity. Based on these studies, a decrease in NAA levels, along with slight improvements of some symptoms and more normal myelination, was reported in patients treated with lithium citrate [17, 251, 252]. The mechanism behind NAA reduction by lithium is unknown, although it has been suggested to prevent NAA release from neurons, increase NAA removal from the brain by affecting permeability of the blood–brain-barrier or from blood by affecting renal excretion [252], or by inhibiting the NAA-synthesis pathway [19]. The anti-epileptic drug topiramate was also reported to slow head growth in two CD patients, though the mechanisms remain poorly understood [253].

NAA synthesis has also been targeted as a treatment possibility for CD. This idea has mainly been inspired by studies showing that CD mice lacking one or both *Nat8L* alleles display less severe spongiform leukodystrophy and neuronal loss [220, 221, 225]. However, complete ablation of N-acetyl synthetase activity is likely undesirable as well. Indeed, *Nat8L* knock-out mice brain display a reduced amount of sphingomyelin and sulfatide [240]. Another study reported that *Nat8L* knock-out mice, have reduced myelin basic protein (MBP) level in the prefrontal cortex in juveniles (but not adults), and exhibited several behavioral deficits, which could be ameliorated by feeding glyceryl triacetate [254]. Various other papers have also indicated neurological issues associated with the *Nat8L* knock-out mice [41, 255–257]. Consequently, if a NAA synthesis inhibition strategy is pursued, reduction rather than complete ablation of NAT8L should be considered. Towards that goal, an adeno-associated viral vector carrying a short harpin RNA against *Nat8L* has been used to suppress spongiform leukodystrophy in neonatal CD mice [258]. Small molecule inhibitors against the NAA synthetase are also being investigated [18, 259, 260].

### Gene therapy for treatment of CD

Of course, the ideal treatment for CD would be to restore ASPA functionality in the brain. Various efforts have been put toward this endeavor. Enzyme replacement therapy using purified ASPA, PEGylated to reduce immunostimulation and increase half-life, has been tested on mice [261], inspired by similar approaches used on the enzyme phenylalanine hydroxylase [262]. A follow-up paper was published a few years later, showing the PEGylation increased diffusion of ASPA from capillaries to surrounding tissues, and exhibited reduced immunogenicity [182].

Currently, the most intensely investigated and arguably best chance at curing CD is through the use of gene therapy to restore intracellular ASPA synthesis, ideally to oligodendrocytes, specifically. Indeed, the nature of CD makes it a good target for gene therapy as the severity of the disease, justifies the risks associates with the treatment, which currently entails creating one [23] or multiple [21, 191] burr holes to deliver the treatment directly into the brain.

Gene therapy involving a non–viral lipid-entrapped, polycation-condensed delivery system was used to deliver an adeno-associated virus (AAV)–based plasmids encoding recombinant *ASPA*. The therapy was used on two CD children (19 months and 24 months old, respectively), following promising data on HEK293 cells, Fischer rats and cynomolgus monkeys. The treatment was well tolerated and did lead to some clinical improvements and reduced NAA levels in the patients, although the NAA levels rose in the months after the therapy, indicating a drop in exogenous ASPA expression over time [23]. A later paper suggested the limited effects and transiency of the aforementioned trial – as well as a larger trial using the same approach (I.N.D.-7307)—to be primarily due to inadequacies of the vector or delivery system, instead advocating for AAV capsids as delivery vectors [191].

In 2003, an AAV vector encoding ASPA was tested on CD mice, showing reduced NAA levels and less spongyform degeneration. However, the vector did not achieve widespread CNS transduction, as areas distal to the injection sites were unaffected by the treatment [263].

Soon thereafter, an AAV2 vector expressing ASPA was tested on tremor rats, lowering their NAA levels and improving their balance and locomotion performance (as measured by a rotarod test) [264]. Other ASPA gene therapy trials in rats include successful treatment of absence-like seizures using adenovirus [265] and the use of a chimeric rAAV1/2 system, which restored ASPA activity for up to 6 months, reduced NAA levels and rescued the seizure phenotype, but did not affect gross brain pathology, such as dilated ventricles and spongiform vacuolization [42].

Following these tests, clinical trials on humans using an AAV2 vector was performed, reporting minimal systemic signs of inflammation or immune stimulation in all subjects [21]. Although a subset of the subjects (3 out of 10), were found to have, low to moderately high levels of AAV2 neutralizing antibodies relative to baseline [21], a follow-up study found no long-term adverse events related to the AAV2 vector [24], while demonstrating a long-lasting reduction in NAA levels and slowed progression of brain atrophy [24].

Further advancement came with the utilization of glia-specific promotors, to induce ASPA expression in glia cells. In 2013, it was shown that the GFAP promoter is highly specific for astrocytes following vector infusion to the brain of neonates and adult mice. In contrast, the MBP promotor, although unspecific in neonates, was specific for oligodendrocytes in 10 days old mice [266]. Later on, these promotors were used in mice along with AAV-vectors to restore ASPA activity specifically in astrocytes [226] and oligodendrocytes [41], respectively.

Likewise, microRNA (miRNA)-mediated post-transcriptional detargeting was used in one study to limit ASPA expression in off-target cells [107]. This process involves the inclusion of miRNA-binding sites in the ASPA-encoding cassette, which are targeted by miRNA specific to peripheral tissues, thus limiting the expression in these tissues.

AAV vectors with tropisms for oligodendrocytes (Olig001) have also been developed [267], and tested on neonatal CD mice [229], and 6 weeks old CD mice [268], which exhibit more progressed CD symptoms similar to what is observed clinically. It should be noted, however, that despite the improved binding to oligodendrocytes, transduction of other cell types still occurs [268]. While both viral tropism and choice of promoter should be optimized for the targeted cell type to maximize gene therapy efficacy, some expression in other tissues may not pose any issues or be neglectable compared to the benefits. At the very least, restoring ASPA activity in astrocytes of CD mice, seems to cure the disease without any apparent downsides [226].

Seeing the potential of reducing NAA-synthesis as a treatment of CD, a 2022 study opted for a combined gene therapy treatment that expressed ASPA and knocked down *Nat8L*, demonstrating its ability to reverse CD in 12 weeks old mice and advocating for its potential in treating more progressed cases of CD [269]. A recent human gene therapy trial involves a rAAV9 to deliver transgene *ASPA* regulated by a modified chicken β-actin (CB6) promoter [270]. Notably, the trail entails simultaneous systemic and intracerebroventricular injections, and the immunosuppressive drugs (Rituximab and Sirolimus) to prevent an immune response against AVV.

Currently (December 2023), two clinical trials on CD patients are ongoing (ClinicalTrials.gov Identifier: NCT04998396 and NCT04833907). NCT04833907 involves a single dose, intracerebroventricular injection using the olig001 vector capsid in up to 24 CD children, whereas NCT04998396 uses an intravenous injection of AAV9-vector to deliver transgene ASPA with a ubiquitous promoter to induce ASPA expression in both neuronal and non-neuronal cell types in up to 18 patients. One issue of gene therapy-based treatment of CD is to get widespread effect in the whole CNS, which likely is necessary to restore normal brain function. Consequently, the AAV9 is of particular interest as it has been shown to pass the blood brain barrier in mice, leading to widespread transduction of the CNS, while simultaneously being less invasive than intracranial delivery [211, 271].

Another promising approach to cure CD is the use of human induced pluripotent stem cells combined with either lentiviral integration of functional *ASPA* alleles to compliment or, more ideally,—homologous recombination to correct—nonfunctional *ASPA* alleles. Using this approach, induced pluripotent stem cells (iPSC) from CD patient fibroblasts, were engineered to express wild-type ASPA and differentiated into either neuronal progenitor cells (NPCs) or oligodendrocyte progenitor cells (OPCs) and then engrafted into immunodeficient *Rag2*^−/−^ CD mice [22, 272]. Later experiments included the development of hypoimmunogenic human iPSC-derived OPCs [273]. If successful, these cells could be used as universal donors to treat CD patients, eliminating the requirement for autologous CD patient cells, inducing pluripotency, reconstituting ASPA expression and differentiating the cells into OPCs. At the time of writing, however, no clinical trials on CD patients using iPSC with reconstituted ASPA are ongoing, although the principle has been applied to CD mice [274]. One challenge of the stem cell approach might be to ensure ASPA activity is restored evenly throughout the brain, which is arguably more feasible with conventional gene therapy. While zones of reconstituted ASPA activity might be sufficient to deal with the elevated NAA levels, this might be insufficient to completely cure CD. Likewise, when validating the efficacy of gene therapies, one should note that urinary NAA levels, although easily obtained, may not reflect the severity of the disease [275]. Thus, urinary NAA levels should be accompanied with other measurements when assessing the efficacy of a given *ASPA* gene therapy treatment.

While reconstitution of functional ASPA activity resolves the cause of the disease, it does not miraculously fix the secondary pathologies of CD such as demyelination and vacuolization. Hence, like many other genetic diseases [276], early intervention is pivotal if the CD patient is to maintain normal development.

This point has been emphasized though multiple studies: In CD mice, initiating triheptanoin treatment in juvenile mice (28 days old) instead of neonates resulted in markedly more modest beneficial effects, indicating a window of therapeutic intervention that corresponds with developmental myelination [15]. Late onset of treatment was also pointed out as the most likely explanation for better effects of treatment with glyceryl triacetate in CD mice compared to CD patients [244]. Likewise, the results of a single dose rAAV vector ASPA gene therapy treatment of CD mice, correlated with treatment time, with injections as late as postnatal day 20 achieving efficacious and sustained improvements [107]. Human gene therapy studies only corroborate this point: In the first CD gene therapy trial, stronger effects of treatment were observed in the 19 months old patient vs the 24 months old [23]. The notion was verified in the later trial where it was noted that, although the therapy changed radiographical disease progression, when given between the first 4–83 months of age, the greatest improvements were seen in subjects treated within the first two years of life. The authors put the ideal window of intervention at the range of 0–3 months [24].

### Diagnosis of Canavan disease

With promising prospects for a gene therapy-based cure of CD, and new trials currently ongoing, an important hurdle to overcome seems to be the timely diagnosis of CD, as to administer the therapy within the optimal time window. Prenatal testing for CD can be done by enzyme activity assays, amniocentesis (followed by measurements of NAA levels) or genetic testing. ASPA activity has been measured in amniocytes or chorionic villi (obtained from chorionic villus sampling) [277, 278].

Activity assays based on amniocytes have been applied [279], but is not recommended due to the low activity found in control amniocytes [278, 280, 281]. Rather, chorionic villi should be used as their ASPA activity is 10 times higher than in amniocytes, and comparable to the levels observed in fibroblasts (which were originally used to assay ASPA activity) [280]. Due to risk of false negative results and maternal contamination of the chorionic villus samples, activity assay should be accompanied by amniocentesis to estimated NAA levels [280].

Amniocentesis can be taken between weeks 16–18 [280] to determine amniotic fluid N-acetylaspartic acid levels [278]. But this is not always perfect either, as slightly elevated NAA levels are harder to interpret [278, 282].

Consequently, DNA diagnosis is the method of choice for prenatal diagnosis of Canavan disease [180, 278, 282–284], ideally with the use of polymorphic markers to rule out maternal cell contamination [278, 284]. However, assuming detection of the disease early postnatally is sufficiently early to enable timely intervention of gene therapy, increased urinary NAA is a reliable marker for CD, especially to distinguish it from other leukodystrophies [88]. Indeed, when looking at newer publications on CD, diagnosis of the disease is determined by either detection of elevated NAA in urine or blood, magnetic resonance spectroscopy (MRS), magnetic resonance imaging (MRI), or clinical symptoms indicative of CD. Most often, genetic testing is used as well to confirm the diagnosis [74, 270, 285].

Notably, DNA diagnosis can be streamlined with DNA-based screenings of other monogenic diseases, thus eliminating the need for specialized equipment and personnel for detecting NAA levels. Additionally, improvements in sequencing techniques and reduced costs further favor the use of DNA based diagnosis of CD patients. Notably, DNA based diagnosis also enables carrier screening of parents and preconception counseling. This combined with in vitro fertilization allows the selection of healthy zygotes.

According to the Canavan foundation website (https://www.canavanfoundation.org/), genetic screenings for CD was historically recommended to Ashkenazi Jewish descendants and families with a history of CD. In the Western world, screening of newborns have been used for decades to test for between 1 and 30 conditions [286] and over the years, the list of diseases tested for through expanded carrier screening (ECS) [287] and newborn screening (NBS) [288] has been growing. Thus, considering the severity of CD and the promising prospects of a gene therapy-based cure, it seems plausible that screening for CD on a wider scale may be implemented in the future.

### Prevalence of pathogenic *ASPA* variants

Due to low disease allele frequencies and a recessive inheritance pattern, Canavan disease has a low prevalence. However, within the Ashkenazi Jewish population, carrier frequencies are much higher, corresponding to a frequency of 1:38 to 1:82, depending on the source [19, 34, 105, 289–295]. Amongst this population approximately 97–98% of the disease-causing alleles are attributed to the alleles E285A (~ 83%) and Y231X (~ 14%) [19, 34, 73, 88, 293, 296], indicating a founder effect within this population [11, 34, 297]. On a smaller scale, another founder effect in an Indian community due to population bottleneck and isolation has also been reported, involving the G176S variant [298].

**Fig. 6:**
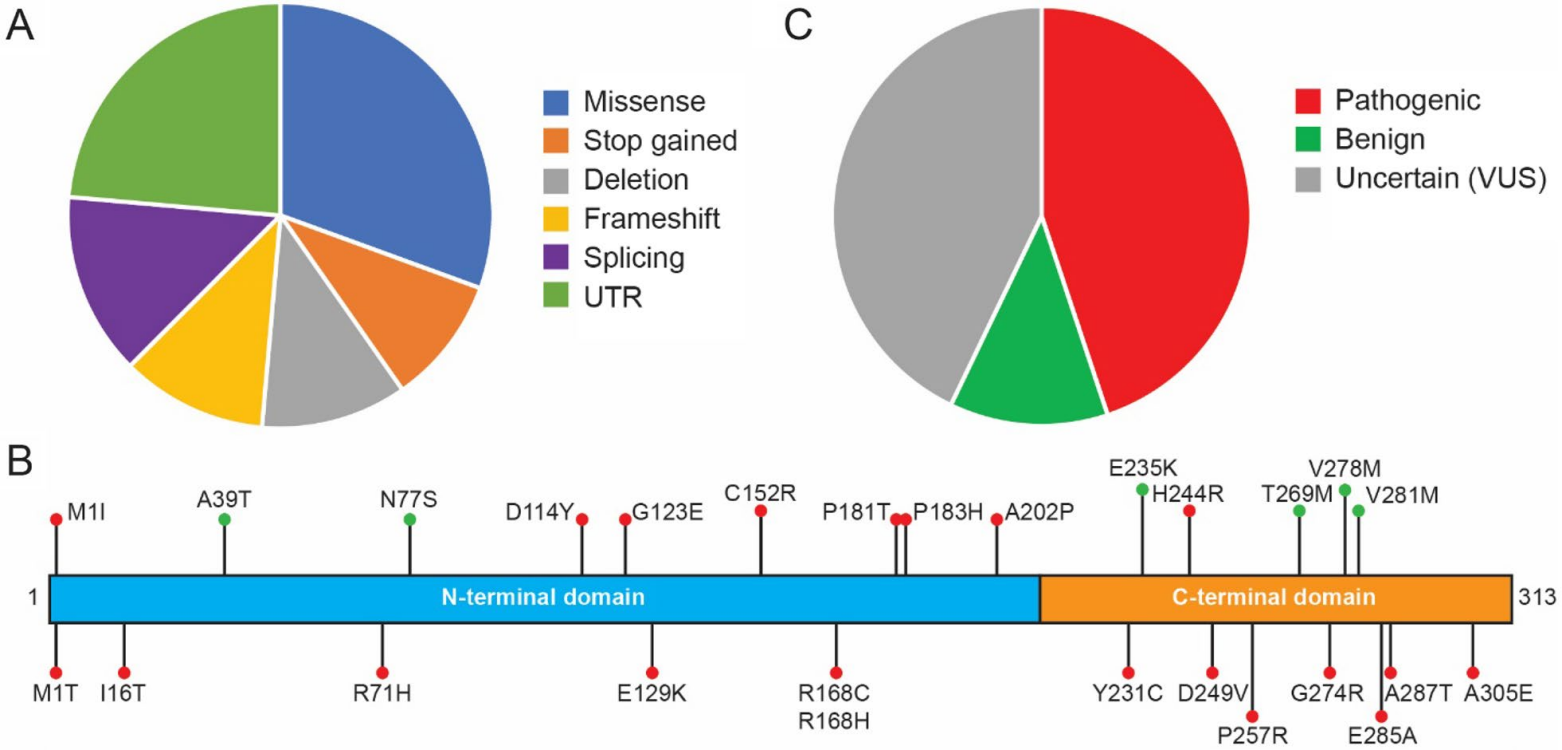
*Reported ASPA gene variants*. **A** The pathogenic and likely pathogenic *ASPA* variants reported in Simple ClinVar (https://simple-clinvar.broadinstitute.org/) [302] distributed between the indicated types of variants. Note that the missense variants represent the largest single class of variants. **B** Localization of the pathogenic and likely-pathogenic (red) missense variants and benign (green) missense variants on the ASPA primary structure. Note that these are roughly evenly distributed and do not cluster to specific regions. **C** Of the missense variants, most (45%) are pathogenic or likely pathogenic (red), while 12% are benign or likely benign (green), and the remaining (43%) are variants of uncertain significance (VUS) (grey)

Outside the Ashkenazi Jewish population, pathogenic variants often arise due to de novo mutations or are confined to single families or small geographical areas [93, 299]. However, the European associated A305E variant is common, accounting for about 40% of all disease alleles in the non-Ashkenazi Jewish population [34, 43, 73, 93, 297, 299–301]. As of now, according to the Simple ClinVar database [302] (accessed December 2023), 72 pathogenic or likely pathogenic ASPA variants are known. Of these, 22 (31%) are missense variants, while the remainder are deletions, frameshifts, splice variants, etc. (Fig. 6A). The missense variants are spread throughout the ASPA coding region (Fig. 6B), consistent with the majority leading to a structural destabilization of the ASPA protein [25, 52]. Finally, when focusing on non-synonymous coding variants, most ASPA variants are pathogenic (61%), while only 8% are benign, and the remainder (31%) are variants of uncertain significance (VUS) (Fig. 6C).

### Genotype–phenotype correlations

In recent years, the increased speed and reduced costs of DNA sequencing has led to a dramatic surge in the number of observed gene variants. Although most genetic variants are likely to be harmless, there is often insufficient evidence to classify newly observed variants as being pathogenic or benign, and these are therefore often designated as variants of uncertain significance (VUS) [303, 304]. In the case of *ASPA*, 33 gene variants are currently classified as VUS in the Simple ClinVar database (accessed June 2023) [302, 305]. The gnomAD database (accessed June 2023) [306] reports 324 *ASPA* gene variants with either conflicting, unknown or without clinical classification. These large numbers of VUS pose a problem for diagnosis and genetic counseling of individuals or families that carry these and yet unknown variants. Moreover, with the promise of gene therapy, incomplete clinical classification of *ASPA* variants may constitute a barrier in the way of treatment.

Since gene variants that result in deletions, frame shifts, early stop codons or that affect mRNA splicing will typically result in dramatic changes in the encoded protein, these can often readily be assigned as pathogenic. However, in case of missense variants, where one amino acid residue is exchanged with another, the variant effects may be more subtle, but can range from increased activity to complete loss of function. Thus, genotype–phenotype predictions for missense variants are often more challenging and accordingly missense variants account for 21 of the 33 currently classified VUS in Simple ClinVar.

**Fig. 7:**
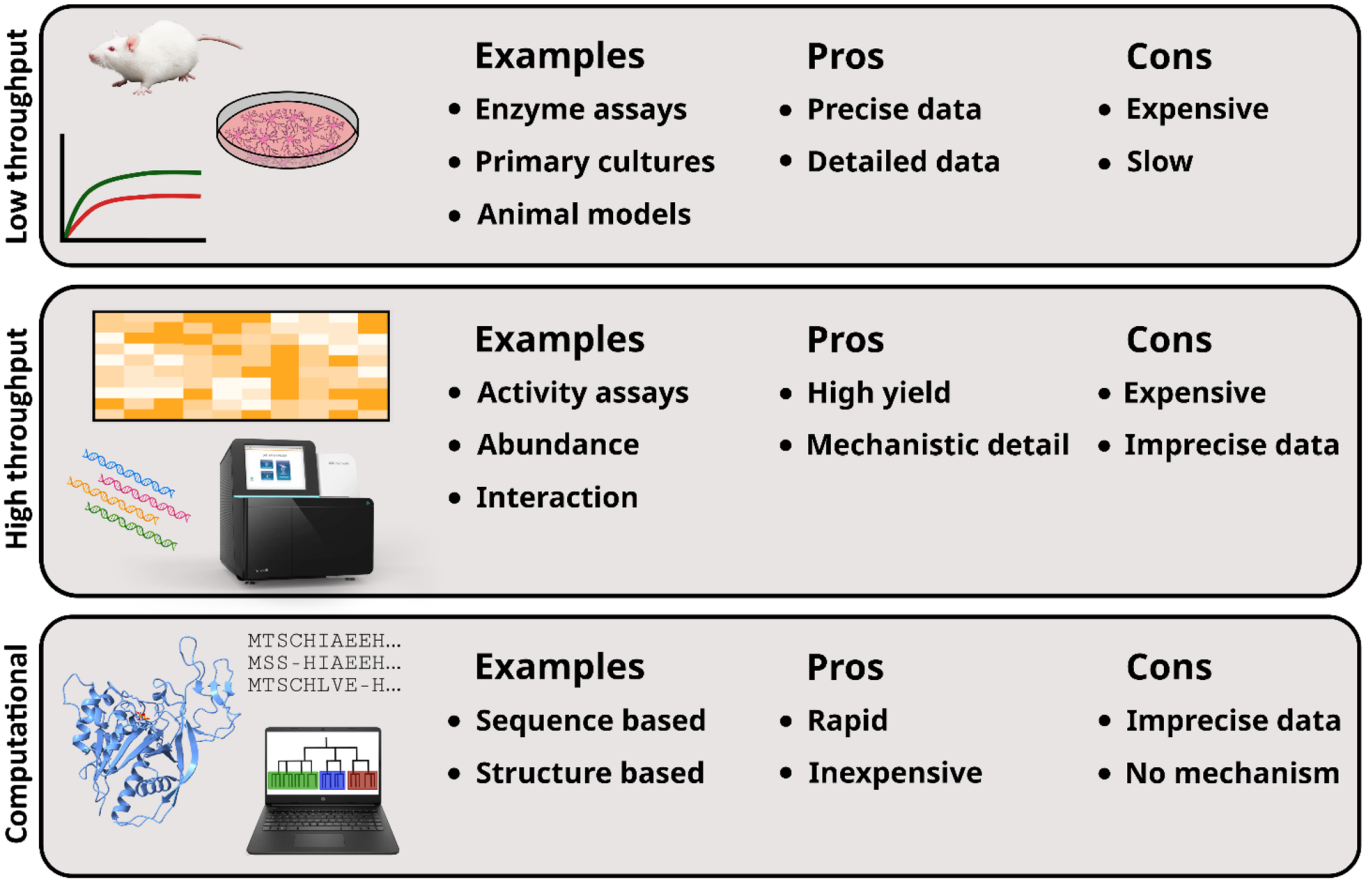
*Methods for probing genotype–phenotype correlations.* Genotype–phenotype correlations can be based on three different types of experimental setups, each with their own pros and cons. (Upper panel) In low throughput experiments, selected variants can be analyzed e.g. by animal studies, in primary cultures or by in vitro enzymatic and/or biophysical assays on purified protein. The results of such experiments are typically highly detailed and precise. However, determining variant effects in this manner is typically time consuming and expensive, and accordingly only a limited number of variants can be assessed in this manner. (Middel panel) Recent developments have allowed for high throughput measurements of variant effects. These so-called MAVEs typically probe either enzyme activity, protein abundance or protein–protein interactions. The advantages of these approaches are that they can inform on thousands of gene variants and depending on type of the assay, they can also provide mechanistic detail (e.g. reduced variant abundance indicates that the variant causes a reduced structural stability). However, in comparison with the low throughput analyses, the obtained results are less precise. (Lower panel) Computational predictors of variant effects typically rely on phylogenetic conservation or structural data. They are rapid and scalable to millions of variants. However, they do not typically provide mechanistic detail and are still imperfect predictors of pathogenicity. Figure compiled using Inkscape (v1.3). Parts of the figure were made using BioRender.com and PyMOL (v2.5.2) using the PDB entry 2O4H [44]

Traditionally, clinical classification of VUS would be assessed in a one-by-one manner, involving meticulous laboratory and animal studies, yielding highly accurate results, but also often detailed mechanistic insights on why a certain variant results in the observed phenotype (Fig. 7). However, considering the number of observed gene variants, this approach is not feasible in terms of time and costs. Recent developments in sequencing and molecular cell biology have allowed rapid functional assessment of all possible, both known and yet unknown, gene variants. These so-called deep mutational scanning (DMS) or multiplexed assays of variant effects (MAVE) assays (Fig. 7) [304, 307–311], along with continuous improvements in computational predictions [312–315], have shown great promise for VUS classification for a range of monogenic diseases, including CD [25].

Using the variant abundance by massively parallel sequencing (VAMP-seq) technology [311], we recently determined the relative abundance of 6152 out of the 6260 (~ 98%) possible single-site missense and nonsense ASPA variants in cultured human cells [25]. Combined with computational predictions based on the phylogenetic conservation and structure of ASPA protein, the results showed that many pathogenic ASPA protein variants are structurally unstable, thus rendering them susceptible to the intracellular protein quality control and degradation systems which in turn leads to insufficient amounts of ASPA protein. Accordingly, those VUS and yet unidentified ASPA variants that are observed at low abundance are likely to be pathogenic. Of the missense VUS currently listed in ClinVar, about 50% display a low abundance [25]. Conversely, those ASPA variants that were found at normal levels can be either pathogenic or benign, and with protein abundance as the sole read out it will not be possible to classify these without testing the enzymatic activity of the variants.

In recent years we have seen tremendous progress in computational tools for prediction of variant effects. In comparison with studies on individual variants and high throughput technologies, the computational tools provide a quick, inexpensive and scalable alternative (Fig. 7) [316].

An early computational variant effect predictor that is still used today, is Sorting Intolerant From Tolerant (SIFT) [317], which is based on sequence homology. Since mutations occur randomly in all species and harmful variants are removed from the gene pool, variants at conserved positions in multiple sequence alignments (MSAs) of orthologous proteins are more likely to be pathogenic than variants at non-conserved positions. Accordingly, sequence conservation is fundamental to most computational variant effect predictors. We recently applied Global Epistatic Model for predicting Mutational Effects (GEMME) [313] to ASPA [25]. Like SIFT, GEMME is based on MSAs, but also accounts for co-variation of amino acid pairs and proves efficient in identifying CD-linked ASPA variants [25, 313]. Another recent MSA-based tool is the Evolutionary model of Variant Effect (EVE), which is based on unsupervised deep learning trained on amino acid sequences of over 140,000 species [312]. The EVE website (https://evemodel.org/, accessed March 2024) includes saturated predictions for ASPA and correctly assigns 14/17 pathogenic or likely-pathogenic and 3/4 benign or likely benign ClinVar missense ASPA variants. On the same set of variants, another recent predictor, AlphaMissense [315], misclassifies two other variants. Since those two variants (P257R and G274R) display very low abundance in the described VAMP-seq analyses [25], this reflects how DMS combined with computational predictors may result in very high accuracies.

While the computational variant effect predictors have become highly accurate, they are still imperfect. In addition, most computational tools do not provide mechanistic insight. Thus, they only provide information on *which* variants are likely pathogenic, and do not inform on *why* a particular variant is harmful. This is because residues that are conserved in MSAs can be conserved because they are critical for e.g. catalysis or substrate binding, but also for folding and stability of the protein structure. Parallel computational predictions of a protein’s structural stability using tools such as FoldX [318] or Rosetta [319] that use the crystal structure of the protein as input (rather than an MSA), have shown some success in untangling such effects [320, 321]. However, these structure-based computational protein stability predictors are also imprecise [322], as they for instance do not take into consideration the folding pathway of the protein.

Finally, as mentioned above, MD simulations offer a computationally more demanding approach to variant effect predictions which also provide information on the variant mechanisms and have with varying success been applied to ASPA. Previously, we tested the effect of the pathogenic and low abundance C152W variant with MD simulation and found no substantial difference to wild-type ASPA on the timescales that could be probed [52]. Utilizing that the structure of four ASPA variants (K213E, Y231C, E285A and F295S) has been solved [108], the Nemukhin group applied MD simulations to predict that two pathogenic ASPA variants, Y231C and F295S, may limit access to the active site [323]. This potentially explains the lower enzyme activity measured for these protein variants [63], although the pathogenicity of these variants is likely also explained by very low abundance [25]. Additional computational studies of aspartoacylase variants using MD simulations have been performed by the Zayed group [324–326]. Using MD simulations, they were able to show that the V31F variant caused conformational changes, affecting catalytic residues in the near vicinity (H21, E24, R63), and reducing the structural stability [324]. Later, the same group found the two pathogenic variants P183H and P183L to have consequences for the structure of ASPA [325]. This is in accordance with our VAMP-seq analyses, where all of these variants display reduced abundance [25].

Lastly, in 2017, Doss and Zayed [326] applied MD simulations and molecular docking to investigate the impact on structure and NAA-binding for four variants (K213E, Y231C, E285A and F295S), using the available crystal structures. Two additional variants of unknown significance (I143V and V186D) were investigated for effects on the structure [10, 327]. All variants were distinguishable from wild-type ASPA, and displayed a lowered compactness, reduced number of intramolecular hydrogen bonds, reduced binding to NAA and poorer accommodation of the Zn^2+^ ion, as calculated using the Protein–Ligand Interaction Profiler [328]. These data indicated that K213E and I143V were benign whereas V186D, Y231C, F295S and E285A were pathogenic to various degrees [326]. These data were corroborated by a range of variant effect prediction tools, including PANTHER, PhD-SNP, SIFT, SNAP, and Meta-SNP, the stability prediction tools iStable server [329] and evolutionary conservation using Consurf [330]. These variants, except for K213E, E285A and I143V, display strongly reduced abundances [25], suggesting that they affect ASPA folding and stability.

For additional information of computational tools for variant effect predictions we refer to these recent and excellent reviews [316, 331–333] and note that as more proteins are analyzed by DMS techniques, the computational tools are likely to improve further and may also offer mechanistic insights into the molecular effects of missense variants.

## Outlook and concluding remarks

As mentioned, gene therapy is currently one of the more promising attempts at curing Canavan disease [23, 24]. However, due to the highly progressive nature of CD, gene therapy-based interventions will likely need to be administered early. Accordingly, rapid and accurate clinical assessment of novel *ASPA* variants will be critical, and high-throughput assays and computational approaches to gain comprehensive genotype–phenotype information on all possible *ASPA* variants are therefore warranted. The recent application of the VAMP-seq technology to ASPA revealed that the major molecular mechanism of the ASPA insufficiency in CD is coupled to a reduced structural stability of the ASPA protein variants [25], and the comprehensive dataset can thus be used in the clinical assessment of variants currently annotated as VUS, but also inform on novel variants that have not yet been encountered in population sequencing. However, as these findings effectively categorize CD as a protein misfolding disorder (proteinopathy), this also potentially allows for novel small molecule-based therapeutic approaches. Presumably, many structurally unstable CD-linked missense ASPA variants will still retain some, albeit insufficient, catalytic function, and can thus be categorized as so-called hypomorph alleles. Accordingly, it should in theory be possible to resuscitate such variants by boosting the cellular levels [334, 335]. Potentially, this can be achieved by increasing synthesis, blocking degradation or by stabilizing structurally unstable variants [334], and further studies on potential transcriptional regulation of *ASPA* and the degradation of ASPA missense variants are therefore a priority. Hopefully, the recent characterization of the degradation of ASPA protein variants [25, 52] shows that ASPA is an ideal model substrate for analyzing the cellular PQC system and may inspire additional studies on ASPA protein folding and degradation. As for stabilizers or folding correctors, these could in principle be any small molecule adept at tightly and specifically interacting with native ASPA [335], without interfering with its catalytic function. Upon binding, the stabilizer would lock unstable ASPA variants in the native conformation, thus averting the PQC-linked degradation and resulting in increased levels of functional enzyme. Indeed, such small molecules have been developed for other hereditary protein folding diseases, most notably cystic fibrosis [336].

As we highlight in the above, another area for further research into ASPA and Canavan disease is on the pathophysiological mechanisms of the disease. The main suggested mechanisms of pathogenicity are not mutually exclusive, and likely both contribute to disease progression. Further experiments, including animal studies, may reveal the relative importance of the proposed mechanisms, which in turn may pave the way for better treatments, and we hope the present literature survey may aid the progress of research aimed at deepening our understanding of ASPA and haste the path towards effective treatments of Canavan disease.

## Data Availability

Not applicable.

## Abbreviations

AAV: Adeno-associated virus
ABC: ATP-binding cassette
ACY2: Aminoacylase II
A/O: Astrocyte-oligodendrocyte
ASPA: Aspartoacylase
BAT: Brown adipose tissue
CBF: Cerebral blood flow
CD: Canavan disease
CNS: Central nervous system
CSF: Cerebrospinal fluid
DMS: Deep mutational scanning
ECF: Extracellular fluid
ECS: Expanded carrier screening
EVE: Evolutionary model of variant effect
GEMME: Global epistatic model for predicting mutational effects
GM: Grey matter
GTA: Glyceryl triacetate
HPLC: High-pressure liquid chromatography
iPSC: Induced pluripotent stem cell
MAS: Malate aspartate shuttle
MAVE: Multiplexed assays of variant effects
MBP: Myelin basic protein
MD: Molecular dynamics
miRNA: MicroRNA
MRI: Magnetic resonance imaging
MRS: Magnetic resonance spectroscopy
MSA: Multiple sequence alignment
NAA: N-acetyl-L-aspartic acid
NAAG: N-acetyl-aspartyl glutamate
NAAG2: N-acetylaspartyl-glutamylglutamate
NBS: Newborn screening
NMDA: N-methyl-D-aspartate
NPC: Neuronal progenitor cell
OPC: Oligodendrocyte progenitor cell
PQC: Protein quality control
SIFT: Sorting intolerant from tolerant
SLC: Solute carrier
VAMP-seq: Variant abundance by massively parallel sequencing
VUS: Variants of uncertain significance
WAT: White adipose tissue
WM: White matter

## References

[1] D’Adamo AF, Smith JC, Woiler C, D’Adamo AFJ, Smith JC, Woiler C (1973). The occurrence of N-acetylaspartate amidohydrolase (Aminoacylase II) in the developing rat. J Neurochem.

[2] D’Adamo AFJ, Wertman E, Foster F, Schneider H (1978). A radiochemical assay for N-acetyl-L-aspartate amidohydrolase (EC 35115) and its occurrence in the tissues of the chicken. Life Sci.

[3] Kaul R, Gao GP, Balamurugan K, Matalon R (1993). Cloning of the human aspartoacylase cDNA and a common missense mutation in Canavan disease. Nat Genet.

[4] Birnbaum SM (1955). Amino acid acylases I and II from hog kidney. Methods Enzym.

[5] Birnbaum SM, Levintow L, Kingsley RB, Greenstein JP (1952). Specificity of amino acid acylases. J Biol Chem.

[6] Kaul R, Balamurugan K, Gao GP, Matalon R (1994). Canavan disease: genomic organization and localization of human ASPA to 17p13-ter and conservation of the ASPA gene during evolution. Genomics.

[7] Sommer A, Sass JO (2012). Expression of aspartoacylase (ASPA) and Canavan disease. Gene.

[8] Kirmani BF, Jacobowitz DM, Namboodiri MAA (2003). Developmental increase of aspartoacylase in oligodendrocytes parallels CNS myelination. Dev Brain Res.

[9] Kirmani BF, Jacobowitz DM, Kallarakal AT, Namboodiri MAA (2002). Aspartoacylase is restricted primarily to myelin synthesizing cells in the CNS: therapeutic implications for Canavan disease. Mol Brain Res.

[10] Bitto E, Bingman CA, Wesenberg GE, McCoy JG, Phillips GN (2007). Structure of aspartoacylase, the brain enzyme impaired in Canavan disease. Proc Natl Acad Sci USA.

[11] Baslow MH (2000). Canavan’s spongiform leukodystrophy: a clinical anatomy of a genetic metabolic CNS disease. J Mol Neurosci.

[12] Baslow MH (2002). Evidence supporting a role for N-acetyl-L-aspartate as a molecular water pump in myelinated neurons in the central nervous system an analytical review. Neurochem Int.

[13] Baslow MH, Guilfoyle DN (2009). Are astrocytes the missing link between lack of brain aspartoacylase activity and the spongiform leukodystrophy in Canavan disease?. Neurochem Res.

[14] Namboodiri AMA, Peethambaran A, Mathew R, Sambhu PA, Hershfield J, Moffett JR, Madhavarao CN (2006). Canavan disease and the role of N-acetylaspartate in myelin synthesis. Mol Cell Endocrinol.

[15] Francis JS, Markov V, Leone P (2014). Dietary triheptanoin rescues oligodendrocyte loss, dysmyelination and motor function in the nur7 mouse model of Canavan disease. J Inherit Metab Dis.

[16] Matalon R, Michals-Matalon K (1999). Biochemistry and molecular biology of Canavan disease. Neurochem Res.

[17] Edo Solsona MD, Fernández LL, Boquet EM, Andrés JLP (2012). Lithium citrate as treatment of canavan disease. Clin Neuropharmacol.

[18] Nešuta O, Thomas AG, Alt J, Hin N, Neužilová A, Long S, Tsukamoto T, Rojas C, Wei H, Slusher BS (2021). High throughput screening cascade to identify human aspartate n-acetyltransferase (ANAT) inhibitors for Canavan disease. ACS Chem Neurosci.

[19] Roscoe RB, Elliott C, Zarros A, Baillie GS (2016). Non-genetic therapeutic approaches to Canavan disease. J Neurol Sci.

[20] Miranda CO, Brites P, Sousa MM, Teixeira CA (2013). Advances and pitfalls of cell therapy in metabolic leukodystrophies. Cell Transplant.

[21] McPhee SWJ, Janson CG, Li C, Samulski RJ, Camp AS, Francis J, Shera D, Lioutermann L, Feely M, Freese A, Leone P (2006). Immune responses to AAV in a phase I study for Canavan disease. J Gene Med.

[22] Chao J, Feng L, Ye P, Chen X, Cui Q, Sun G, Zhou T, Tian E, Li W, Hu W, Riggs AD, Matalon R, Shi Y (2022). Therapeutic development for Canavan disease using patient iPSCs introduced with the wild-type ASPA gene. IScience.

[23] Leone P, Janson CG, Bilaniuk L, Wang Z, Sorgi F, Huang L, Matalon R, Kaul R, Zeng Z, Freese A, McPhee SW, Mee E, During MJ (2000). Aspartoacylase gene transfer to the mammalian central nervous system with therapeutic implications for Canavan disease. Ann Neurol.

[24] Leone P, Shera D, McPhee SWJ, Francis JS, Kolodny EH, Bilaniuk LT, Wang D-J, Assadi M, Goldfarb O, Goldman HW, Freese A, Young D, During MJ, Samulski RJ, Janson CG (2012). Long-term follow-up after gene therapy for canavan disease. Sci Transl Med.

[25] Grønbæk-Thygesen M, Voutsinos V, Johansson KE, Schulze TK, Cagiada M, Pedersen L, Clausen L, Nariya S, Powell RL, Stein A, Fowler DM, Lindorff-Larsen K, Hartmann-Petersen R (2023). Deep mutational scanning reveals a tight correlation between protein degradation and toxicity of thousands of non-native aspartoacylase protein variants. BioRxiv.

[26] Hershfield JR, Madhavarao CN, Moffett JR, Benjamins JA, Garbern JY, Namboodiri A (2006). Aspartoacylase is a regulated nuclear-cytoplasmic enzyme. FASEB J.

[27] Kitada K, Akimitsu T, Shigematsu Y, Kondo A, Maihara T, Yokoi N, Kuramoto T, Sasa M, Serikawa T (2000). Accumulation of N-acetyl-L-aspartate in the brain of the tremor rat, a mutant exhibiting absence-like seizure and spongiform degeneration in the central nervous system. J Neurochem.

[28] Matalon R, Rady PL, Platt KA, Skinner HB, Quast MJ, Campbell GA, Matalon K, Ceci JD, Tyring SK, Nehls M, Surendran S, Wei J, Ezell EL, Szucs S (2000). Knock-out mouse for Canavan disease: a model for gene transfer to the central nervous system. J Gene Med.

[29] Klugmann M, Leichtlein CB, Kaplitt MG (2006). Clinical trials of gene therapy for canavan disease. Gene therapy of the central nervous system.

[30] Mersmann N, Tkachev D, Jelinek R, Röth PT, Möbius W, Ruhwedel T, Rühle S, Weber-Fahr W, Sartorius A, Klugmann M (2011). Aspartoacylase-lacZ knockin mice: an engineered model of Canavan disease. PLoS ONE.

[31] The Human Protein ATLAS - ASPA, (n.d.). https://www.proteinatlas.org/ENSG00000108381-ASPA/tissue (Accessed 13 Aug 2022).

[32] Hagenfeldt L, Bollgren I, Venizelos N (1987). N-acetylaspartic aciduria due to aspartoacylase deficiency—a new aetiology of childhood leukodystrophy. J Inherit Metab Dis.

[33] Barash V, Flhor D, Morag B, Boneh A, Elpeleg ON, Gilon C (1991). A radiometric assay for aspartoacylase activity in human fibroblasts: application for the diagnosis of Canavan’s disease. Clin Chim Acta.

[34] Matalon R, Michals K, Kaul R (1995). Canavan disease: from spongy degeneration to molecular analysis. J Pediatr.

[35] Madhavarao CN, Moffett JR, Moore RA, Viola RE, Namboodiri MAA, Jacobowitz DM (2004). Immunohistochemical localization of aspartoacylase in the rat central nervous system. J Comp Neurol.

[36] Moffett JR, Arun P, Ariyannur PS, Garbern J, Jacobowitz DM, Namboodiri AMA (2011). extensive aspartoacylase expression in the rat central nervous. System.

[37] Klugmann M, Symes CW, Klaussner BK, Leichtlein CB, Serikawa T, Young D, During MJ (2003). Identification and distribution of aspartoacylase in the postnatal rat brain. NeuroReport.

[38] Baslow MH, Suckow RF, Sapirstein V, Hungund BL (1999). Expression of aspartoacylase activity in cultured rat macroglial cells is limited to oligodendrocytes. J Mol Neurosci.

[39] Bhakoo KK, Craig TJ, Styles P (2001). Developmental and regional distribution of aspartoacylase in rat brain tissue. J Neurochem.

[40] von Jonquieres G, Froud KE, Klugmann CB, Wong ACY, Housley GD, Klugmann M (2014). Loss of central auditory processing in a mouse model of Canavan disease. PLoS ONE.

[41] von Jonquieres G, Spencer ZHT, Rowlands BD, Klugmann CB, Bongers A, Harasta AE, Parley KE, Cederholm J, Teahan O, Pickford R, Delerue F, Ittner LM, Fröhlich D, McLean CA, Don AS, Schneider M, Housley GD, Rae CD, Klugmann M (2018). Uncoupling N-acetylaspartate from brain pathology: implications for Canavan disease gene therapy. Acta Neuropathol.

[42] Klugmann M, Leichtlein CB, Symes CW, Serikawa T, Young D, During MJ (2005). Restoration of aspartoacylase activity in CNS neurons does not ameliorate motor deficits and demyelination in a model of Canavan disease. Mol Ther.

[43] Hershfield JR, Pattabiraman N, Madhavarao CN, Namboodiri MAA (2007). Mutational analysis of aspartoacylase: implications for Canavan DIsease. Brain Res.

[44] Le Coq J, Pavlovsky A, Malik R, Sanishvili R, Chengfu X, Viola RE (2008). Examination of the mechanism of human brain aspartoacylase through the binding of an intermediate analogue. Biochemistry.

[45] Makarova KS, Grishin NV (1999). The Zn-peptidase superfamily: Functional convergence after evolutionary divergence. J Mol Biol.

[46] Le Coq J, An H-J, Lebrilla C, Viola RE (2006). Characterization of human aspartoacylase: the brain enzyme responsible for Canavan disease. Biochemistry.

[47] Goldstein FB (1976). Amidohydrolases of brain; enzymatic hydrolysis of N-acetyl-L-aspartate and other N-acyl-L-amino acids. J Neurochem.

[48] Kots ED, Lushchekina SV, Varfolomeev SD, Nemukhin AV (2017). Role of protein dimeric interface in allosteric inhibition of N-acetyl-aspartate hydrolysis by human aspartoacylase. J Chem Inf Model.

[49] Moore RA, Le Coq J, Faehnle CR, Viola RE (2003). Purification and preliminary characterization of brain aspartoacylase. Arch Biochem Biophys.

[50] Kots ED, Khrenova MG, Lushchekina SV, Varfolomeev SD, Grigorenko BL, Nemukhin AV (2016). Modeling the complete catalytic cycle of aspartoacylase. J Phys Chem B.

[51] Khrenova MG, Kots ED, Varfolomeev SD, Lushchekina SV, Nemukhin AV (2017). Three faces of N—acetylaspartate: activator, substrate, and inhibitor of human aspartoacylase. J Phys Chem.

[52] Gersing SK, Wang Y, Grønbæk-Thygesen M, Kampmeyer C, Clausen L, Willemoës M, Andréasson C, Stein A, Lindorff-Larsen K, Hartmann-Petersen R (2021). Mapping the degradation pathway of a disease-linked aspartoacylase variant. PLoS Genet.

[53] Badonyi M, Marsh JA (2023). Buffering of genetic dominance by allele-specific protein complex assembly. Sci Adv.

[54] Natan E, Wells JN, Teichmann SA, Marsh JA (2017). Regulation, evolution and consequences of cotranslational protein complex assembly. Curr Opin Struct Biol.

[55] Fleming MC, Lowry OH (1966). The measurement of free and N-acetylated aspartic acids in the nervous system. J Neurochem.

[56] Matalon R, Michals K, Sebesta D, Deanching M, Gashkoff P, Casanova J, Optiz JM, Reynolds JF (1988). Aspartoacylase deficiency and N-acetylaspartic aciduria in patients with canavan disease. Am J Med Genet.

[57] Kaul R, Casanova J, Johnson AB, Tang P, Matalon R (1991). Purification, Characterization, and Localization of Aspartoacylase from Bovine Brain. J Neurochem.

[58] Becker I, Eckhardt M (2023). An enzymatic fluorimetric assay for determination of N-acetylaspartate. Anal Biochem.

[59] Madhavarao CN, Hammer JA, Quarles RH, Namboodiri MAA (2002). A radiometric assay for aspartoacylase activity in cultured oligodendrocytes. Anal Biochem.

[60] Di Pietro V, Gambacurta A, Amorini AM, Finocchiaro A, D’Urso S, Ceccarelli L, Tavazzi B, Giardina B, Lazzarino G (2008). A new T677C mutation of the aspartoacylase gene encodes for a protein with no enzymatic activity. Clin Biochem.

[61] Zeng BJ, Wang ZH, Ribeiro LA, Leone P, De Gasperi R, Kim SJ, Raghavan S, Ong E, Pastores GM, Kolodny EH (2002). Identification and characterization of novel mutations of the aspartoacylase gene in non-Jewish patients with Canavan disease. J Inherit Metab Dis.

[62] Martin M, Labouesse J, Canioni P, Merle M (1993). N-acetyl-L-aspartate and acetate 1H NMR signal overlapping under mild acidic pH conditions. Magn Reson Med.

[63] Zano S, Wijayasinghe YS, Malik R, Smith J, Viola RE (2013). Relationship between enzyme properties and disease progression in Canavan disease. J Inherit Metab Dis.

[64] Mendes MI, Smith DEC, Pop A, Lennertz P, Fernandez Ojeda MR, Kanhai WA, van Dooren SJM, Anikster Y, Barić I, Boelen C, Campistol J, de Boer L, Kariminejad A, Kayserili H, Roubertie A, Verbruggen KT, Vianey-Saban C, Williams M, Salomons GS (2017). Clinically distinct phenotypes of canavan disease correlate with residual aspartoacylase enzyme activity. Hum Mutat.

[65] Canavan MM (1931). Schilder’s encephalitis periaxialis diffusa: report of a case in a child aged sixteen and one-half months. Arch Neurol Psychiatry.

[66] Globus JH, Strauss I (1928). Progressive degenerative subcortical encephalopathy: (Schilder’s disease). Arch Neurol Psychiatry.

[67] Matalon RM, Michals-Matalon K (2000). Spongy degeneration of the brain Canavan disease: biochemical and molecular findings. FBL.

[68] Van Bogaert L, Bertrand I (1949). Sur une idiotie familiale avec degerescence sponglieuse de neuraxe (note preliminaire). Acta Neurol Belg.

[69] Kvittingen EA, Guldal G, Børsting S, Skalpe IO, Stokke O, Jellum E (1986). N-Acetylaspartic aciduria in a child with a progressive cerebral atrophy. Clin Chim Acta.

[70] Divry P, Vianey-Liaud C, Gay C, Macabeo V, Rapin F, Echenne B (1988). N-Acetylaspartic aciduria: report of three new cases in children with a neurological syndrome associating macrocephaly and leukodystrophy. J Inherit Metab Dis.

[71] Kumar S, Mattan NS, de Vellis J (2006). Canavan disease: a white matter disorder. Ment Retard Dev Disabil Res Rev.

[72] Surendran S, Michals-Matalon K, Quast MJ, Tyring SK, Wei J, Ezell EL, Matalon R (2003). Canavan disease: a monogenic trait with complex genomic interaction. Mol Genet Metab.

[73] Kaul R, Gao GP, Aloya M, Balamurugan K, Petrosky A, Michals K, Matalon R (1994). Canavan disease: mutations among jewish and non-Jewish patients. Am J Hum Genet.

[74] Bley A, Denecke J, Kohlschütter A, Schön G, Hischke S, Guder P, Bierhals T, Lau H, Hempel M, Eichler FS (2021). The natural history of Canavan disease: 23 new cases and comparison with patients from literature. Orphanet J Rare Dis.

[75] Hoshino H, Kubota M (2014). Canavan disease: clinical features and recent advances in research. Pediatr Int.

[76] R. Matalon, L. Delgado, K. Michals-Matalon, Canavan Disease., in: M.P. Adam, G.M. Mirzaa, R.A. Pagon, S.E. Wallace, L.J.H. Bean, K.W. Gripp, A. Amemiya (Eds.), Seattle (WA), 1993.

[77] Duray M, Kılavuz G, Altuğ F (2015). Physiotherapy assessment of canavan disease: case report. Int J Ther Appl.

[78] Traeger EC, Rapin I (1998). The clinical course of canavan disease. Pediatr Neurol.

[79] Ozand PT, Gascon GG, Dhalla M (1990). Aspartoacylase deficiency and Canavan disease in Saudi Arabia. Am J Med Genet.

[80] Gowda VK, Bharathi NK, Bettaiah J, Bhat M, Shivappa SK (2021). Canavan disease: clinical and laboratory profile from southern part of India. Ann Indian Acad Neurol.

[81] Matalon R, Michals-Matalon K (1998). Molecular basis of Canavan disease. Eur J Paediatr Neurol.

[82] Zelnik N, Luder AS, Elpeleg ON, Gross-Tsur V, Amir N, Hemli JA, Fattal A, Harel S (1993). Protracted clinical course for patients with Canavan disease. Dev Med Child Neurol.

[83] Adachi M, Schneck L, Cara J, Volk BW (1973). Spongy degeneration of the central nervous system (Van Bogaert and Bertrand type, Canavan’s disease): a review. Hum Pathol.

[84] Mirimanoff P (1976). Hereditary spongiform dystrophy in young children (Canavan: van Bogaert-Bertrand). J Neurol Sci.

[85] Gambetti P, Mellman WJ, Gonatas NK (1969). Familial spongy degeneration of the central nervous system (Van Bogaert-Bertrand disease)—an ultrastructural study. Acta Neuropathol.

[86] Traka M, Wollmann RL, Cerda SR, Dugas J, Barres BA, Popko B (2008). Nur7 is a nonsense mutation in the mouse aspartoacylase gene that causes spongy degeneration of the CNS. J Neurosci.

[87] Pleasure D, Guo F, Chechneva O, Bannerman P, McDonough J, Burns T, Wang Y, Hull V (2020). Pathophysiology and treatment of Canavan disease. Neurochem Res.

[88] Namboodiri AMA, Moffett JR, Arun P, Mathew R, Namboodiri S, Potti A, Hershfield J, Kirmani B, Jacobowitz DM, Madhavarao CN, Moffett JR, Tieman SB, Weinberger DR, Coyle JT, Namboodiri AMA (2006). Defective myelin lipid synthesis as a pathogenic mechanism of canavan disease BT—N-acetylaspartate. Springer.

[89] Adornato BT, O’Brien JS, Lampert PW, Roe TF, Neustein HB (1972). Cerebral spongy degeneration of infancy. a biochemical and ultrastructural study of affected twins. Neurology.

[90] Luo Y, Huang K (1984). Spongy degeneration of the CNS in infancy. Arch Neurol.

[91] Kamoshita S, Rapin I, Suzuki K, Suzuki K (1968). Spongy degeneration of the brain. a chemical study of two cases including isolation and characterization of myelin. Neurology.

[92] Adachi M, Torii J, Schneck L, Volk BW (1972). Electron miscroscopic and enzyme histochemical studies of the cerebellum in spongy degeneration. Acta Neuropathol.

[93] Shaag A, Anikster Y, Christensen E, Glustein JZ, Fois A, Michelakakis H, Nigro F, Pronicka E, Ribes A, Zabot MT, Elpeleg ON (1995). The molecular basis of canavan (aspartoacylase deficiency) disease in European non-Jewish patients. Am J Hum Genet.

[94] Jauhari P, Saini L, Chakrabarty B, Kumar A, Gulati S (2018). Juvenile Canavan disease: a leukodystrophy without white matter changes. Neuropediatrics.

[95] Nguyen HV, Ishak GE (2015). Canavan disease—unusual imaging features in a child with mild clinical presentation. Pediatr Radiol.

[96] Janson CG, Kolodny EH, Zeng BJ, Raghavan S, Pastures G, Torres P, Assadi M, McPhee S, Goldfarb O, Saslow B, Freese A, Wang DJ, Bilaniuk L, Shera D, Leone P (2006). Mild-onset presentation of Canavan’s disease associated with novel G212A point mutation in aspartoacylase gene. Ann Neurol.

[97] Çakar NE, Aksu Uzunhan T (2020). A case of juvenile Canavan disease with distinct pons involvement. Brain Dev.

[98] Mizuguchi K, Hoshino H, Hamaguchi H, Kubota M (2009). Long term clinical course of Canavan disease–a rare Japanese case. No Hattatsu Brain Dev..

[99] Toft PB, Geiss-Holtorff R, Rolland MO, Pryds O, Müller-Forell W, Christensen E, Lehnert W, Lou HC, Ott D, Hennig J (1993). Magnetic resonance imaging in juvenile Canavan disease. Eur J Pediatr.

[100] Zafeiriou DI, Kleijer WJ, Maroupoulos G, Anastasiou AL, Augoustidou-Savvopoulou P, Papadopoulou F, Kontopoulos EE, Fagan E, Payne S (1999). Protracted course of N-acetylaspartic aciduria in two non-Jewish siblings: identical clinical and magnetic resonance imaging findings. Brain Dev.

[101] Tacke U, Olbrich H, Sass JO, Fekete A, Horvath J, Ziyeh S, Kleijer WJ, Rolland MO, Fisher S, Payne S, Vargiami E, Zafeiriou DI, Omran H (2005). Possible genotype-phenotype correlations in children with mild clinical course of Canavan disease. Neuropediatrics.

[102] Yalcinkaya C, Benbir G, Salomons GS, Karaarslan E, Rolland MO, Jakobs C, van der Knaap MS (2005). Atypical MRI findings in Canavan disease: a patient with a mild course. Neuropediatrics.

[103] Velinov M, Zellers N, Styles J, Wisniewski K (2008). Homozygosity for mutation G212A of the gene for aspartoacylase is associated with atypical form of Canavan’s disease. Clin Genet.

[104] Sarret C, Boespflug-Tanguy O, Rodriguez D (2016). Atypical clinical and radiological course of a patient with Canavan disease. Metab Brain Dis.

[105] Elpeleg ON, Anikster Y, Barash V, Branski D, Shaag A (1994). The frequency of the C854 mutation in the aspartoacylase gene in Ashkenazi Jews in Israel. Am J Hum Genet.

[106] Brismar J, Brismar G, Gascon G, Ozand P (1990). Canavan disease: CT and MR imaging of the brain. AJNR Am J Neuroradiol.

[107] Ahmed SS, Li H, Cao C, Sikoglu EM, Denninger AR, Su Q, Eaton S, Liso Navarro AA, Xie J, Szucs S, Zhang H, Moore C, Kirschner DA, Seyfried TN, Flotte TR, Matalon R, Gao G (2013). A single intravenous rAAV injection as late as P20 achieves efficacious and sustained CNS gene therapy in Canavan mice. Mol Ther.

[108] Wijayasinghe YS, Pavlovsky AG, Viola RE (2014). Aspartoacylase catalytic deficiency as the cause of canavan disease: a structural perspective. Biochemistry.

[109] Tallan HH (1957). Studies on the distribution of N-Acetyl-L-aspartic acid in brain. J Biol Chem.

[110] Miyake M, Kakimoto Y, Sorimachi M (1981). A gas chromatographic method for the determination of N-Acetyl-l-aspartic acid, N-Acetyl-α- aspartylglutamic acid and β-Citryl-l-Glutamic acid and their distributions in the brain and other organs of various species of animals. J Neurochem.

[111] Tallan HH, Moore S, Stein WH (1956). N-acetyl-l-aspartic acid in brain. J Biol Chem.

[112] Marcucci F, Mussini E, Valzelli L, Garattini S (1966). distribution of N-Acetyl-l-aspartic acid in rat brain. J Neurochem.

[113] Moffett JR, Ross B, Arun P, Madhavarao CN, Namboodiri AMA (2007). N-Acetylaspartate in the CNS: from neurodiagnostics to neurobiology. Prog Neurobiol.

[114] Baslow MH, Guilfoyle DN (2013). Canavan disease, a rare early-onset human spongiform leukodystrophy: insights into its genesis and possible clinical interventions. Biochimie.

[115] Arun P, Moffett JR, Namboodiri AMA (2009). Evidence for mitochondrial and cytoplasmic N-acetylaspartate synthesis in SH-SY5Y neuroblastoma cells. Neurochem Int.

[116] Ariyannur PS, Madhavarao CN, Namboodiri AMA (2008). N-acetylaspartate synthesis in the brain: mitochondria vs microsomes. Brain Res.

[117] Lu Z-H, Chakraborty G, Ledeen RW, Yahya D, Wu G (2004). N-Acetylaspartate synthase is bimodally expressed in microsomes and mitochondria of brain. Brain Res Mol Brain Res.

[118] Madhavarao CN, Chinopoulos C, Chandrasekaran K, Namboodiri MAA (2003). Characterization of the N-acetylaspartate biosynthetic enzyme from rat brain. J Neurochem.

[119] Truckenmiller ME, Namboodiri MAA, Brownstein MJ, Neale JH (1985). N-Acetylation of L-Aspartate in the nervous system: differential distribution of a specific enzyme. J Neurochem.

[120] Hofer DC, Zirkovits G, Pelzmann HJ, Huber K, Pessentheiner AR, Xia W, Uno K, Miyazaki T, Kon K, Tsuneki H, Pendl T, Al Zoughbi W, Madreiter-Sokolowski CT, Trausinger G, Abdellatif M, Schoiswohl G, Schreiber R, Eisenberg T, Magnes C, Sedej S, Eckhardt M, Sasahara M, Sasaoka T, Nitta A, Hoefler G, Graier WF, Kratky D, Auwerx J, Bogner-Strauss JG (2019). N-acetylaspartate availability is essential for juvenile survival on fat-free diet and determines metabolic health. FASEB J Off Publ Fed Am Soc Exp Biol..

[121] Taylor DL, Davies SE, Obrenovitch TP, Urenjak J, Richards DA, Clark JB, Symon L (1994). Extracellular N-acetylaspartate in the rat brain: in vivo determination of basal levels and changes evoked by high K+. J Neurochem.

[122] Baslow MH (1999). Molecular water pumps and the aetiology of Canavan disease: a case of the sorcerer’s apprentice. J Inherit Metab Dis.

[123] Wei H, Moffett JR, Amanat M, Fatemi A, Tsukamoto T, Namboodiri AM, Slusher BS (2022). The pathogenesis of, and pharmacological treatment for, Canavan disease. Drug Discov Today.

[124] Jansen RS, Mahakena S, de Haas M, Borst P, van de Wetering K (2015). ATP-binding cassette subfamily C member 5 (ABCC5) functions as an efflux transporter of glutamate conjugates and analogs. J Biol Chem.

[125] Morris HB, David NG (2016). Evidence that N-acetylaspartylglutamate is the astrocyte-targeted neurovascular coupling agent that regulates slow tonic control of brain blood flow. J. Glycomics Metab.

[126] Tranberg M, Stridh MH, Guy Y, Jilderos B, Wigström H, Weber SG, Sandberg M (2004). NMDA-receptor mediated efflux of N-acetylaspartate: physiological and/or pathological importance?. Neurochem Int.

[127] Shah AJ, de la Flor R, Atkins A, Slone-Murphy J, Dawson LA (2008). Development and application of a liquid chromatography/tandem mass spectrometric assay for measurement of N-acetylaspartate, N-acetylaspartylglutamate and glutamate in brain slice superfusates and tissue extracts. J Chromatogr B Anal Technol Biomed Life Sci.

[128] Zollinger M, Amsler U, Do KQ, Streit P, Cuénod M (1988). Release of N-acetylaspartylglutamate on depolarization of rat brain slices. J Neurochem.

[129] Sager TN, Fink-Jensen A, Hansen AJ (1997). Transient elevation of interstitial N-acetylaspartate in reversible global brain ischemia. J Neurochem.

[130] Sarchielli P, Tarducci R, Presciutti O, Gobbi G, Pelliccioli GP, Stipa G, Alberti A, Capocchi G (2005). Functional 1H-MRS findings in migraine patients with and without aura assessed interictally. Neuroimage.

[131] Baslow MH, Cain CK, Sears R, Wilson DA, Bachman A, Gerum S, Guilfoyle DN (2016). Stimulation-induced transient changes in neuronal activity, blood flow and N-acetylaspartate content in rat prefrontal cortex: a chemogenetic fMRS-BOLD study. NMR Biomed.

[132] Landim RCG, Edden RAE, Foerster B, Li LM, Covolan RJM, Castellano G (2016). Investigation of NAA and NAAG dynamics underlying visual stimulation using MEGA-PRESS in a functional MRS experiment. Magn Reson Imaging.

[133] Manzhurtsev A, Menschchikov P, Yakovlev A, Ublinskiy M, Bozhko O, Kupriyanov D, Akhadov T, Varfolomeev S, Semenova N (2021). 3T MEGA-PRESS study of N-acetyl aspartyl glutamate and N-acetyl aspartate in activated visual cortex. MAGMA.

[134] Baslow MH, Guilfoyle DN (2002). Effect of N-acetylaspartic acid on the diffusion coefficient of water: a proton magnetic resonance phantom method for measurement of osmolyte-obligated water. Anal Biochem.

[135] Baslow MH, Hrabe J, Guilfoyle DN (2007). Dynamic relationship between neurostimulation and N-acetylaspartate metabolism in the human visual cortex: evidence that NAA functions as a molecular water pump during visual stimulation. J Mol Neurosci.

[136] Sugahara K, Jianying Z, Kodama H (1994). Liquid chromatographic—mass spectrometric analysis of N-acetylamino acids in human urine. J Chromatogr B Biomed Sci Appl.

[137] Moreno A, Ross BD, Blüml S (2001). Direct determination of the N-acetyl-L-aspartate synthesis rate in the human brain by (13)C MRS and [1-(13)C]glucose infusion. J Neurochem.

[138] Fujita T, Katsukawa H, Yodoya E, Wada M, Shimada A, Okada N, Yamamoto A, Ganapathy V (2005). Transport characteristics of N-acetyl-L-aspartate in rat astrocytes: Involvement of sodium-coupled high-affinity carboxylate transporter NaC3/NaDC3-mediated transport system. J Neurochem.

[139] Huang W, Wang H, Kekuda R, Fei YJ, Friedrich A, Wang J, Conway SJ, Cameron RS, Leibach FH, Ganapathy V (2000). Transport of N-acetylaspartate by the Na(+)-dependent high-affinity dicarboxylate transporter NaDC3 and its relevance to the expression of the transporter in the brain. J Pharmacol Exp Ther.

[140] Long PM, Moffett JR, Namboodiri AMA, Viapiano MS, Lawler SE, Jaworski DM (2013). N-acetylaspartate (NAA) and N-acetylaspartylglutamate (NAAG) promote growth and inhibit differentiation of glioma stem-like cells. J Biol Chem.

[141] George RL, Huang W, Naggar HA, Smith SB, Ganapathy V (2004). Transport of N-acetylaspartate via murine sodium/dicarboxylate cotransporter NaDC3 and expression of this transporter and aspartoacylase II in ocular tissues in mouse. Biochim Biophys Acta.

[142] Wang Y, Hull V, Sternbach S, Popovich B, Burns T, McDonough J, Guo F, Pleasure D (2021). Ablating the transporter sodium-dependent dicarboxylate transporter 3 prevents leukodystrophy in canavan disease mice. Ann Neurol.

[143] Bai X, Chen X, Feng Z, Hou K, Zhang P, Fu B, Shi S (2006). Identification of basolateral membrane targeting signal of human sodium-dependent dicarboxylate transporter 3. J Cell Physiol.

[144] Orthmann-Murphy JL, Abrams CK, Scherer SS (2008). Gap junctions couple astrocytes and oligodendrocytes. J Mol Neurosci.

[145] Nagy JI, Ionescu A-V, Lynn BD, Rash JE (2003). Coupling of astrocyte connexins Cx26, Cx30, Cx43 to oligodendrocyte Cx29, Cx32, Cx47: Implications from normal and connexin32 knockout mice. Glia.

[146] Menichella DM, Goodenough DA, Sirkowski E, Scherer SS, Paul DL (2003). Connexins are critical for normal myelination in the CNS. J Neurosci Off J Soc Neurosci.

[147] Bergoffen J, Scherer SS, Wang S, Scott MO, Bone LJ, Paul DL, Chen K, Lensch MW, Chance PF, Fischbeck KH (1993). Connexin mutations in X-linked charcot-marie-tooth disease. Science.

[148] Tress O, Maglione M, Zlomuzica A, May D, Dicke N, Degen J, Dere E, Kettenmann H, Hartmann D, Willecke K (2011). Pathologic and phenotypic alterations in a mouse expressing a connexin47 missense mutation that causes pelizaeus-merzbacher-like disease in humans. PLoS Genet.

[149] Odermatt B, Wellershaus K, Wallraff A, Seifert G, Degen J, Euwens C, Fuss B, Büssow H, Schilling K, Steinhäuser C, Willecke K (2003). Connexin 47 (Cx47)-deficient mice with enhanced green fluorescent protein reporter gene reveal predominant oligodendrocytic expression of Cx47 and display vacuolized myelin in the CNS. J Neurosci Off J Soc Neurosci.

[150] Magnotti LM, Goodenough DA, Paul DL (2011). Deletion of oligodendrocyte Cx32 and astrocyte Cx43 causes white matter vacuolation, astrocyte loss and early mortality. Glia.

[151] Yan HD, Ishihara K, Serikawa T, Sasa M (2003). Activation by N-acetyl-L-aspartate of acutely dissociated hippocampal neurons in rats via metabotropic glutamate receptors. Epilepsia.

[152] Neale JH, Bzdega T, Wroblewska B (2000). N-Acetylaspartylglutamate: the most abundant peptide neurotransmitter in the mammalian central nervous system. J Neurochem.

[153] Neale JH, Olszewski RT, Zuo D, Janczura KJ, Profaci CP, Lavin KM, Madore JC, Bzdega T (2011). Advances in understanding the peptide neurotransmitter NAAG and appearance of a new member of the NAAG neuropeptide family. J Neurochem.

[154] Vallianatou T, Lin W, Bèchet NB, Correia MS, Shanbhag NC, Lundgaard I, Globisch D (2021). Differential regulation of oxidative stress, microbiota-derived, and energy metabolites in the mouse brain during sleep. J Cereb Blood Flow Metab Off J Int Soc Cereb Blood Flow Metab.

[155] Moffett JR, Namboodiri MA (1995). Differential distribution of N-acetylaspartylglutamate and N-acetylaspartate immunoreactivities in rat forebrain. J Neurocytol.

[156] Lodder-Gadaczek J, Becker I, Gieselmann V, Wang-Eckhardt L, Eckhardt M (2011). N-acetylaspartylglutamate synthetase II synthesizes N-acetylaspartylglutamylglutamate. J Biol Chem.

[157] Morland C, Nordengen K (2022). N-Acetyl-Aspartyl-Glutamate in brain health and disease. Int J Mol Sci.

[158] Nordengen K, Morland C, Slusher BS, Gundersen V (2020). Dendritic Localization and Exocytosis of NAAG in the rat Hippocampus. Cereb Cortex.

[159] Neale JH (2011). N-acetylaspartylglutamate is an agonist at mGluR3 in vivo and in vitro. J Neurochem.

[160] Williamson LC, Neale JH (1988). Ultrastructural localization of N-acetylaspartylglutamate in synaptic vesicles of retinal neurons. Brain Res.

[161] Walder KK, Ryan SB, Bzdega T, Olszewski RT, Neale JH, Lindgren CA (2013). Immunohistological and electrophysiological evidence that N-acetylaspartylglutamate is a co-transmitter at the vertebrate neuromuscular junction. Eur J Neurosci.

[162] Mh B (2017). The bimodal nature of neurovascular coupling: slow tonic and rapid phasic responses are separately controlled by specific astrocyte metabotropic and ionotropic glutamate receptors. J Mol Genet Med.

[163] Bzdega T, Turi T, Wroblewska B, She D, Chung HS, Kim H, Neale JH (1997). Molecular cloning of a peptidase against N-acetylaspartylglutamate from a rat hippocampal cDNA library. J Neurochem.

[164] Bzdega T, Crowe SL, Ramadan ER, Sciarretta KH, Olszewski RT, Ojeifo OA, Rafalski VA, Wroblewska B, Neale JH (2004). The cloning and characterization of a second brain enzyme with NAAG peptidase activity. J Neurochem.

[165] Collard F, Stroobant V, Lamosa P, Kapanda CN, Lambert DM, Muccioli GG, Poupaert JH, Opperdoes F, Van Schaftingen E (2010). Molecular identification of N-acetylaspartylglutamate synthase and beta-citrylglutamate synthase. J Biol Chem.

[166] Sácha P, Zámecník J, Barinka C, Hlouchová K, Vícha A, Mlcochová P, Hilgert I, Eckschlager T, Konvalinka J (2007). Expression of glutamate carboxypeptidase II in human brain. Neuroscience.

[167] Inglese M, Rusinek H, George IC, Babb JS, Grossman RI, Gonen O (2008). Global average gray and white matter N-acetylaspartate concentration in the human brain. Neuroimage.

[168] Krukowski P, Podgórski P, Guziński M, Szewczyk P, Sąsiadek M (2010). Analysis of the brain proton magnetic resonance spectroscopy—differences between normal grey and white matter. Polish J Radiol.

[169] Birken DL, Oldendorf WH (1989). N-Acetyl-L-aspartic acid: a literature review of a compound prominent in 1H-NMR spectroscopic studies of brain. Neurosci Biobehav Rev.

[170] Tackley G, Kong Y, Minne R, Messina S, Winkler A, Cavey A, Everett R, DeLuca GC, Weir A, Craner M, Tracey I, Palace J, Stagg CJ, Emir U (2021). An In-vivo 1H-MRS short-echo time technique at 7T: quantification of metabolites in chronic multiple sclerosis and neuromyelitis optica brain lesions and normal appearing brain tissue. Neuroimage.

[171] Chiew M, Jiang W, Burns B, Larson P, Steel A, Jezzard P, Albert Thomas M, Emi UE (2017). Density-weighted concentric rings k-space trajectory for (1) H magnetic resonance spectroscopic imaging at 7 T. NMR Biomed.

[172] Nassirpour S, Chang P, Henning A (2018). High and ultra-high resolution metabolite mapping of the human brain using 1H FID MRSI at 9.4T. Neuroimage.

[173] Fotuhi M, Standaert DG, Testa CM, Penney JBJ, Young AB (1994). Differential expression of metabotropic glutamate receptors in the hippocampus and entorhinal cortex of the rat. Brain Res Mol Brain Res.

[174] Clark JB (1998). N-acetyl aspartate: a marker for neuronal loss or mitochondrial dysfunction. Dev Neurosci.

[175] Liemburg E, Sibeijn-Kuiper A, Bais L, Pijnenborg G, Knegtering H, van der Velde J, Opmeer E, de Vos A, Dlabac-De Lange J, Wunderink L (2016). Prefrontal NAA and Glx levels in different stages of psychotic disorders: a 3T 1H-MRS study. Sci Rep.

[176] Pessentheiner AR, Pelzmann HJ, Walenta E, Schweiger M, Groschner LN, Graier WF, Kolb D, Uno K, Miyazaki T, Nitta A, Rieder D, Prokesch A, Bogner-Strauss JG (2013). NAT8L (N-acetyltransferase 8-like) accelerates lipid turnover and increases energy expenditure in brown adipocytes. J Biol Chem.

[177] Prokesch A, Pelzmann HJ, Pessentheiner AR, Huber K, Madreiter-Sokolowski CT, Drougard A, Schittmayer M, Kolb D, Magnes C, Trausinger G, Graier WF, Birner-Gruenberger R, Pospisilik JA, Bogner-Strauss JG (2016). N-acetylaspartate catabolism determines cytosolic acetyl-CoA levels and histone acetylation in brown adipocytes. Sci Rep.

[178] Surendran S, Matalon KM, Szucs S, Tyring SK, Matalon R (2003). Metabolic changes in the knockout mouse for Canavan’s disease: implications for patients with Canavan’s disease. J Child Neurol.

[179] Madhavarao CN, Arun P, Moffett JR, Szucs S, Surendran S, Matalon R, Garbern J, Hristova D, Johnson A, Jiang W, Namboodiri MAA (2005). Defective N-acetylaspartate catabolism reduces brain acetate levels and myelin lipid synthesis in Canavan’s disease. Proc Natl Acad Sci U S A.

[180] Matalon R, Kaul R, Michals K (1993). Canavan disease: biochemical and molecular studies. J Inherit Metab Dis.

[181] Clark JF, Doepke A, Filosa JA, Wardle RL, Lu A, Meeker TJ, Pyne-Geithman GJ (2006). N-acetylaspartate as a reservoir for glutamate. Med Hypotheses.

[182] Poddar NK, Zano S, Natarajan R, Yamamoto B, Viola RE (2014). Enhanced brain distribution of modified aspartoacylase. Mol Genet Metab.

[183] Nguyen T, Kirsch BJ, Asaka R, Nabi K, Quinones A, Tan J, Antonio MJ, Camelo F, Li T, Nguyen S, Hoang G, Nguyen K, Udupa S, Sazeides C, Shen Y-A, Elgogary A, Reyes J, Zhao L, Kleensang A, Chaichana KL, Hartung T, Betenbaugh MJ, Marie SK, Jung JG, Wang T-L, Gabrielson E, Le A (2019). Uncovering the role of N-Acetyl-Aspartyl-glutamate as a glutamate reservoir in cancer. Cell Rep.

[184] Baslow MH, Resnik TR (1997). Canavan disease. analysis of the nature of the metabolic lesions responsible for development of the observed clinical symptoms. J Mol Neurosci.

[185] Moffett JR, Puthillathu N, Vengilote R, Jaworski DM, Namboodiri AM (2020). acetate revisited: a key biomolecule at the nexus of metabolism, epigenetics and oncogenesis-part 1: acetyl-coa, acetogenesis and Acyl-CoA short-chain synthetases. Front Physiol.

[186] Baslow MH, Suckow RF, Berg MJ, Marks N, Saito M, Bhakoo KK (2001). Differential expression of carnosine, homocarnosine and N-acetyl-L-histidine hydrolytic activities in cultured rat macroglial cells. J Mol Neurosci.

[187] Sass JO, Mohr V, Olbrich H, Engelke U, Horvath J, Fliegauf M, Loges NT, Schweitzer-Krantz S, Moebus R, Weiler P, Kispert A, Superti-Furga A, Wevers RA, Omran H (2006). Mutations in ACY1, the gene encoding aminoacylase 1, cause a novel inborn error of metabolism. Am J Hum Genet.

[188] Yamada S, Tanaka Y, Sameshima M, Furuichi M (1993). Properties of Nα-acetylhistidine deacetylase in brain of rainbow trout Oncorhynchus mykiss. Comp Biochem Physiol Part B Biochem.

[189] Blüml S (1999). In vivo quantitation of cerebral metabolite concentrations using natural abundance 13C MRS at 1.5 T. J Magn Reson.

[190] Barker PB, Bryan RN, Kumar AJ, Naidu S (1992). Proton NMR spectroscopy of Canavan’s disease. Neuropediatrics.

[191] Janson C, McPhee S, Bilaniuk L, Haselgrove J, Testaiuti M, Freese A, Wang D-J, Shera D, Hurh P, Rupin J, Saslow E, Goldfarb O, Goldberg M, Larijani G, Sharrar W, Liouterman L, Camp A, Kolodny E, Samulski J, Leone P (2002). Clinical protocol. gene therapy of canavan disease: AAV-2 vector for neurosurgical delivery of aspartoacylase gene (ASPA) to the human brain. Hum Gene Ther.

[192] Kelley RI, Stamas JN (1992). Quantification of N-acetyl-L-aspartic acid in urine by isotope dilution gas chromatography-mass spectrometry. J Inherit Metab Dis.

[193] Burlina AP, Ferrari V, Divry P, Gradowska W, Jakobs C, Bennett MJ, Sewell AC, Dionisi-Vici C, Burlina AB (1999). N-acetylaspartylglutamate in Canavan disease: an adverse effector?. Eur J Pediatr.

[194] Tortorella C, Ruggieri M, Monte E, Ceci DE, Iaffaldano P, Direnzo V, Mastrapasqua M, Frigeri A, Amato MP, Hakiki B, Ghezzi A, Lugaresi A, De Luca G, Patti F, Damico E, Sola P, Simone AM, Svelto M, Livrea P, Trojano M (2011). Serum and CSF N-acetyl aspartate levels differ in multiple sclerosis and neuromyelitis optica. J Neurol Neurosurg Psychiatry.

[195] Jakobs C, ten Brink HJ, Langelaar SA, Zee T, Stellaard F, Macek M, Srsnová K, Srsen S, Kleijer WJ (1991). Stable isotope dilution analysis of N-acetylaspartic acid in CSF, blood, urine and amniotic fluid: accurate postnatal diagnosis and the potential for prenatal diagnosis of Canavan disease. J Inherit Metab Dis.

[196] Surendran S, Rady PL, Michals-Matalon K, Quast MJ, Rassin DK, Campbell GA, Ezell EL, Wei J, Tyring SK, Szucs S, Matalon R (2003). Expression of glutamate transporter, GABRA6, serine proteinase inhibitor 2 and low levels of glutamate and GABA in the brain of knock-out mouse for Canavan disease. Brain Res Bull.

[197] Kaya N, Imtiaz F, Colak D, Al-Sayed M, Al-Odaib A, Al-Zahrani F, Al-Mubarak BR, Al-Owain M, Al-Dhalaan H, Chedrawi A, Al-Hassnan Z, Coskun S, Sakati N, Ozand P, Meyer BF (2008). Genome-wide gene expression profiling and mutation analysis of Saudi patients with Canavan disease. Genet Med Off J Am Coll Med Genet.

[198] Zhou Y, Danbolt NC (2014). Glutamate as a neurotransmitter in the healthy brain. J Neural Transm.

[199] Akimitsu T, Kurisu K, Hanaya R, Iida K, Kiura Y, Arita K, Matsubayashi H, Ishihara K, Kitada K, Serikawa T, Sasa M (2000). Epileptic seizures induced by N-acetyl-L-aspartate in rats: in vivo and in vitro studies. Brain Res.

[200] Mochel F, Boildieu N, Barritault J, Sarret C, Eymard-Pierre E, Seguin F, Schiffmann R, Boespflug-Tanguy O (2010). Elevated CSF N-acetylaspartylglutamate suggests specific molecular diagnostic abnormalities in patients with white matter diseases. Biochim Biophys Acta.

[201] Fonnum F (1984). Glutamate: a neurotransmitter in mammalian brain. J Neurochem.

[202] Neale JH, Yamamoto T (2020). N-acetylaspartylglutamate (NAAG) and glutamate carboxypeptidase II: An abundant peptide neurotransmitter-enzyme system with multiple clinical applications. Prog Neurobiol.

[203] Karaman S, Barnett J, Sykes GP, Hong B, Delaney B (2011). Two-generation reproductive and developmental toxicity assessment of dietary N-acetyl-L-aspartic acid in rats. Food Chem Toxicol.

[204] Appu AP, Moffett JR, Arun P, Moran S, Nambiar V, Krishnan JKS, Puthillathu N, Namboodiri AMA (2017). Increasing N-acetylaspartate in the brain during postnatal myelination does not cause the CNS pathologies of Canavan Disease. Front Mol Neurosci.

[205] Feng L, Chao J, Zhang M, Pacquing E, Hu W, Shi Y (2023). Developing a human iPSC-derived three-dimensional myelin spheroid platform for modeling myelin diseases. IScience.

[206] Baslow MH (1999). The existence of molecular water pumps in the nervous system: a review of the evidence. Neurochem Int.

[207] Baslow MH (2003). Brain N-acetylaspartate as a molecular water pump and its role in the etiology of Canavan disease: a mechanistic explanation. J Mol Neurosci.

[208] Baslow MH (1998). Function of the N-acetyl-L-histidine system in the vertebrate eye. Evidence in support of a role as a molecular water pump. J Mol Neurosci.

[209] Meinild A, Klaerke DA, Loo DD, Wright EM, Zeuthen T (1998). The human Na+-glucose cotransporter is a molecular water pump. J Physiol.

[210] Loo DD, Zeuthen T, Chandy G, Wright EM (1996). Cotransport of water by the Na+/glucose cotransporter. Proc Natl Acad Sci U S A.

[211] Ahmed SS, Gao G (2015). Making the white matter matters: progress in understanding canavan’s disease and therapeutic interventions through eight decades. JIMD Rep.

[212] D’Adamo AF, Yatsu FM (1966). Acetate metabolism in the nervous system. n-acetyl-l-aspartic acid and the biosynthesis of brain lipids. J Neurochem.

[213] D’Adamo AFJ, Gidez LI, Yatsu FM (1968). Acetyl transport mechanisms. Involvement of N-acetyl aspartic acid in de novo fatty acid biosynthesis in the developing rat brain. Exp Brain Res.

[214] Burri R, Steffen C, Herschkowitz N (1991). N-acetyl-L-aspartate is a major source of acetyl groups for lipid synthesis during rat brain development. Dev Neurosci.

[215] Mehta V, Namboodiri MA (1995). N-acetylaspartate as an acetyl source in the nervous system. Brain Res Mol Brain Res.

[216] Chakraborty G, Mekala P, Yahya D, Wu G, Ledeen RW (2001). Intraneuronal N-acetylaspartate supplies acetyl groups for myelin lipid synthesis: Evidence for myelin-associated aspartoacylase. J Neurochem.

[217] Jacobson KB (1959). Studies on the role of N-acetylaspartic acid in mammalian brain. J Gen Physiol.

[218] Wiggins RC (1982). Myelin development and nutritional insufficiency. Brain Res.

[219] Watson RE, Desesso JM, Hurtt ME, Cappon GD (2006). Postnatal growth and morphological development of the brain: a species comparison. Birth Defects Res B Dev Reprod Toxicol.

[220] Guo F, Bannerman P, Mills Ko E, Miers L, Xu J, Burns T, Li S, Freeman E (2015). JA McDonough D pleasure, ablating N-acetylaspartate prevents leukodystrophy in a Canavan disease model. Ann Neurol.

[221] Maier H, Wang-Eckhardt L, Hartmann D, Gieselmann V, Eckhardt M (2015). N-acetylaspartate synthase deficiency corrects the myelin phenotype in a canavan disease mouse model but does not affect survival time. J Neurosci.

[222] Martin E, Capone A, Schneider J, Hennig J, Thiel T (2001). Absence of N-acetylaspartate in the human brain: impact on neurospectroscopy?. Ann Neurol.

[223] Boltshauser E, Schmitt B, Wevers RA, Engelke U, Burlina AB, Burlina AP (2004). Follow-up of a child with hypoacetylaspartia. Neuropediatrics.

[224] Burlina AP, Schmitt B, Engelke U, Wevers RA, Burlina AB, Boltshauser E, Moffett JR, Tieman SB, Weinberger DR, Coyle JT, Namboodiri AMA (2006). Hypoacetylaspartia: clinical and biochemical follow-up of a patient bt—n-acetylaspartate. Springer.

[225] Sohn J, Bannerman P, Guo F, Burns T, Miers L, Croteau C, Singhal NK, McDonough JA, Pleasure D (2017). Suppressing N-Acetyl-l-aspartate synthesis prevents loss of neurons in a murine model of Canavan leukodystrophy. J Neurosci Off J Soc Neurosci.

[226] Gessler DJ, Li D, Xu H, Su Q, Sanmiguel J, Tuncer S, Moore C, King J, Matalon R, Gao G (2017). Redirecting N-acetylaspartate metabolism in the central nervous system normalizes myelination and rescues Canavan disease. JCI Insight.

[227] Arun P, Madhavarao CN, Moffett JR, Hamilton K, Grunberg NE, Ariyannur PS, Gahl WA, Anikster Y, Mog S, Hallows WC, Denu JM, Namboodiri AMA (2010). Metabolic acetate therapy improves phenotype in the tremor rat model of Canavan disease. J Inherit Metab Dis.

[228] Francis JS, Strande L, Markov V, Leone P (2012). Aspartoacylase supports oxidative energy metabolism during myelination. J Cereb. Blood Flow Metab Off J Int Soc Cereb Blood Flow Metab..

[229] Francis JS, Wojtas I, Markov V, Gray SJ, McCown TJ, Samulski RJ, Bilaniuk LT, Wang D-J, De Vivo DC, Janson CG, Leone P (2016). N-acetylaspartate supports the energetic demands of developmental myelination via oligodendroglial aspartoacylase. Neurobiol Dis.

[230] Mathew R, Arun P, Madhavarao CN, Moffett JR, Namboodiri MAA (2005). Progress toward acetate supplementation therapy for Canavan disease: glyceryl triacetate administration increases acetate, but not N-acetylaspartate, levels in brain. J Pharmacol Exp Ther.

[231] Holeček M (2023). Aspartic acid in health and disease. Nutrients.

[232] Pardo B, Contreras L, Choi I-Y, Gruetter R (2012). Redox shuttles in the brain. Neural Metab.

[233] Jalil MA, Begum L, Contreras L, Pardo B, Iijima M, Li MX, Ramos M, Marmol P, Horiuchi M, Shimotsu K, Nakagawa S, Okubo A, Sameshima M, Isashiki Y, Del Arco A, Kobayashi K, Satrústegui J, Saheki T (2005). Reduced N-acetylaspartate levels in mice lacking aralar, a brain- and muscle-type mitochondrial aspartate-glutamate carrier. J Biol Chem.

[234] Satrústegui J, Pardo B, Del Arco A (2007). Mitochondrial transporters as novel targets for intracellular calcium signaling. Physiol Rev.

[235] Satrústegui J, Contreras L, Ramos M, Marmol P, del Arco A, Saheki T, Pardo B (2007). Role of aralar, the mitochondrial transporter of aspartate-glutamate, in brain N-acetylaspartate formation and Ca(2+) signaling in neuronal mitochondria. J Neurosci Res.

[236] Wibom R, Lasorsa FM, Töhönen V, Barbaro M, Sterky FH, Kucinski T, Naess K, Jonsson M, Pierri CL, Palmieri F, Wedell A (2009). AGC1 deficiency associated with global cerebral hypomyelination. N Engl J Med.

[237] Copray S, Huynh JL, Sher F, Casaccia-Bonnefil P, Boddeke E (2009). Epigenetic mechanisms facilitating oligodendrocyte development, maturation, and aging. Glia.

[238] Ye F, Chen Y, Hoang T, Montgomery RL, Zhao X, Bu H, Hu T, Taketo MM, van Es JH, Clevers H, Hsieh J, Bassel-Duby R, Olson EN, Lu QR (2009). HDAC1 and HDAC2 regulate oligodendrocyte differentiation by disrupting the beta-catenin-TCF interaction. Nat Neurosci.

[239] Kumar S, Biancotti JC, Matalon R, de Vellis J (2009). Lack of aspartoacylase activity disrupts survival and differentiation of neural progenitors and oligodendrocytes in a mouse model of Canavan disease. J Neurosci Res.

[240] Singhal NK, Huang H, Li S, Clements R, Gadd J, Daniels A, Kooijman EE, Bannerman P, Burns T, Guo F, Pleasure D, Freeman E, Shriver L, McDonough J (2017). The neuronal metabolite NAA regulates histone H3 methylation in oligodendrocytes and myelin lipid composition. Exp Brain Res.

[241] Lotun A, Li D, Xu H, Su Q, Tuncer S, Sanmiguel J, Mooney M, Baer CE, Ulbrich R, Eyles SJ, Strittmatter L, Hayward LJ, Gessler DJ, Gao G (2023). Renewal of oligodendrocyte lineage reverses dysmyelination and CNS neurodegeneration through corrected N-acetylaspartate metabolism. Prog Neurobiol.

[242] Irilouzadian R, Goudarzi A, Hesami H, Sarmadian R, Biglari HN, Gilani A (2023). An unusual case of a toddler with Canavan disease with frequent intractable seizures: a case report and review of the literature. SAGE Open Med Case Rep.

[243] Madhavarao CN, Arun P, Anikster Y, Mog SR, Staretz-Chacham O, Moffett JR, Grunberg NE, Gahl WA, Namboodiri AMA (2009). Glyceryl triacetate for Canavan disease: a low-dose trial in infants and evaluation of a higher dose for toxicity in the tremor rat model. J Inherit Metab Dis.

[244] Segel R, Anikster Y, Zevin S, Steinberg A, Gahl WA, Fisher D, Staretz-Chacham O, Zimran A, Altarescu G (2011). A safety trial of high dose glyceryl triacetate for Canavan disease. Mol Genet Metab.

[245] Leaf DE, Goldfarb DS (2007). Mechanisms of action of acetazolamide in the prophylaxis and treatment of acute mountain sickness. J Appl Physiol.

[246] Reiss WG, Oles KS (1996). Acetazolamide in the treatment of seizures. Ann Pharmacother.

[247] S. Bluml, K. Seymour, M. Philippart, R. Matalon, B. Ross. Elevated brain water in Canavan Disease: impact of a diuretic therapy. Intl Soc Magn Res 171. 1998.

[248] O’Donnell T, Rotzinger S, Nakashima TT, Hanstock CC, Ulrich M, Silverstone PH (2000). Chronic lithium and sodium valproate both decrease the concentration of myo-inositol and increase the concentration of inositol monophosphates in rat brain. Brain Res.

[249] Baslow MH, Suckow RF, Hungund BL (2000). Effects of ethanol and of alcohol dehydrogenase inhibitors on the reduction of N-acetylaspartate levels of brain in mice in vivo: a search for substances that may have therapeutic value in the treatment of Canavan disease. J Inherit Metab Dis.

[250] Baslow MH, Kitada K, Suckow RF, Hungund BL, Serikawa T (2002). The effects of lithium chloride and other substances on levels of brain N-acetyl-L-aspartic acid in Canavan disease-like rats. Neurochem Res.

[251] Assadi M, Janson C, Wang DJ, Goldfarb O, Suri N, Bilaniuk L, Leone P (2010). Lithium citrate reduces excessive intra-cerebral N-acetyl aspartate in Canavan disease. Eur J Paediatr Neurol.

[252] Janson CG, Assadi M, Francis J, Bilaniuk L, Shera D, Leone P (2005). Lithium citrate for Canavan disease. Pediatr Neurol.

[253] Topçu M, Yalnizoğlu D, Saatçi I, Haliloğlu G, Topaloğlu H, Senbil N, Onol S, Coşkun T (2004). Effect of topiramate on enlargement of head in Canavan disease: a new option for treatment of megalencephaly. Turk J Pediatr.

[254] Sumi K, Uno K, Noike H, Tomohiro T, Hatanaka Y, Furukawa-Hibi Y, Nabeshima T, Miyamoto Y, Nitta A (2017). Behavioral impairment in SHATI/NAT8L knockout mice via dysfunction of myelination development. Sci Rep.

[255] Wulaer B, Kunisawa K, Hada K, Jaya Suento W, Kubota H, Iida T, Kosuge A, Nagai T, Yamada K, Nitta A, Yamamoto Y, Saito K, Mouri A, Nabeshima T (2021). Shati/Nat8l deficiency disrupts adult neurogenesis and causes attentional impairment through dopaminergic neuronal dysfunction in the dentate gyrus. J Neurochem.

[256] Toriumi K, Tanaka J, Mamiya T, Alkam T, Kim H-C, Nitta A, Nabeshima T (2018). Shati/Nat8l knockout mice show behavioral deficits ameliorated by atomoxetine and methylphenidate. Behav Brain Res.

[257] Furukawa-Hibi Y, Nitta A, Fukumitsu H, Somiya H, Toriumi K, Furukawa S, Nabeshima T, Yamada K (2012). Absence of SHATI/Nat8l reduces social interaction in mice. Neurosci Lett.

[258] Bannerman P, Guo F, Chechneva O, Burns T, Zhu X, Wang Y, Kim B, Singhal NK, McDonough JA, Pleasure D (2018). Brain Nat8l knockdown suppresses spongiform leukodystrophy in an aspartoacylase-deficient Canavan disease mouse model. Mol Ther.

[259] Mutthamsetty V, Dahal GP, Wang Q, Viola RE (2020). Development of bisubstrate analog inhibitors of aspartate N-acetyltransferase, a critical brain enzyme. Chem Biol Drug Des.

[260] Thangavelu B, Mutthamsetty V, Wang Q, Viola RE (2017). Design and optimization of aspartate N-acetyltransferase inhibitors for the potential treatment of Canavan disease, Bioorganic. Med Chem.

[261] Zano S, Malik R, Szucs S, Matalon R, Viola RE (2011). Modification of aspartoacylase for potential use in enzyme replacement therapy for the treatment of Canavan disease. Mol Genet Metab.

[262] Wang L, Gamez A, Sarkissian CN, Straub M, Patch MG, Han GW, Striepeke S, Fitzpatrick P, Scriver CR, Stevens RC (2005). Structure-based chemical modification strategy for enzyme replacement treatment of phenylketonuria. Mol Genet Metab.

[263] Matalon R, Surendran S, Rady PL, Quast MJ, Campbell GA, Matalon KM, Tyring SK, Wei J, Peden CS, Ezell EL, Muzyczka N, Mandel RJ (2003). Adeno-associated virus-mediated aspartoacylase gene transfer to the brain of knockout mouse for canavan disease. Mol Ther.

[264] McPhee SWJ, Francis J, Janson CG, Serikawa T, Hyland K, Ong EO, Raghavan SS, Freese A, Leone P (2005). Effects of AAV-2-mediated aspartoacylase gene transfer in the tremor rat model of Canavan disease. Brain Res Mol Brain Res.

[265] Seki T, Matsubayashi H, Amano T, Kitada K, Serikawa T, Sakai N, Sasa M (2002). Adenoviral gene transfer of aspartoacylase into the tremor rat, a genetic model of epilepsy, as a trial of gene therapy for inherited epileptic disorder. Neurosci Lett.

[266] von Jonquieres G, Mersmann N, Klugmann CB, Harasta AE, Lutz B, Teahan O, da Housley G, Fröhlich D, Krämer-Albers EM, Klugmann M (2013). Glial promoter selectivity following AAV-delivery to the immature brain. PLoS ONE.

[267] Powell SK, Khan N, Parker CL, Samulski RJ, Matsushima G, Gray SJ, McCown TJ (2016). Characterization of a novel adeno-associated viral vector with preferential oligodendrocyte tropism. Gene Ther.

[268] Francis JS, Markov V, Wojtas ID, Gray S, McCown T, Samulski RJ, Figueroa M, Leone P (2021). Preclinical biodistribution, tropism, and efficacy of oligotropic AAV/Olig001 in a mouse model of congenital white matter disease. Mol Ther Methods Clin Dev.

[269] Fröhlich D, Kalotay E, von Jonquieres G, Bongers A, Lee B, Suchowerska AK, Housley GD, Klugmann M (2022). Dual-function AAV gene therapy reverses late-stage Canavan disease pathology in mice. Front Mol Neurosci.

[270] Corti M, Byrne BJ, Gessler DJ, Thompson G, Norman S, Lammers J, Coleman KE, Liberati C, Elder ME, Escolar ML, Tuna IS, Mesaros C, Kleiner GI, Barbouth DS, Gray-Edwards HL, Clement N, Cleaver BD, Gao G (2023). Adeno-associated virus-mediated gene therapy in a patient with Canavan disease using dual routes of administration and immune modulation. Mol Ther Methods Clin Dev.

[271] Foust KD, Nurre E, Montgomery CL, Hernandez A, Chan CM, Kaspar BK (2009). Intravascular AAV9 preferentially targets neonatal neurons and adult astrocytes. Nat Biotechnol.

[272] Feng L, Chao J, Tian E, Li L, Ye P, Zhang M, Chen X, Cui Q, Sun G, Zhou T, Felix G, Qin Y, Li W, Meza ED, Klein J, Ghoda L, Hu W, Luo Y, Dang W, Hsu D, Gold J, Goldman SA, Matalon R, Shi Y (2020). Cell-based therapy for canavan disease using human iPSC-derived NPCs and OPCs. Adv Sci.

[273] Feng L, Chao J, Ye P, Luong Q, Sun G, Liu W, Cui Q, Flores S, Jackson N, Shayento ANH, Sun G, Liu Z, Hu W, Shi Y (2023). Developing hypoimmunogenic human iPSC-derived oligodendrocyte progenitor cells as an off-the-shelf cell therapy for myelin disorders. Adv Sci.

[274] Surendran S, Shihabuddin LS, Clarke J, Taksir TV, Stewart GR, Parsons G, Yang W, Tyring SK, Michals-Matalon K, Matalon R (2004). Mouse neural progenitor cells differentiate into oligodendrocytes in the brain of a knockout mouse model of Canavan disease. Brain Res Dev Brain Res.

[275] Di Pietro V, Cavallari U, Amorini AM, Lazzarino G, Longo S, Poggiani C, Cavalli P, Tavazzi B (2013). New T530C mutation in the aspartoacylase gene caused Canavan disease with no correlation between severity and N-acetylaspartate excretion. Clin Biochem.

[276] Gray SJ (2016). Timing of gene therapy interventions: the earlier, the better. Mol Ther.

[277] Bennett MJ, Gibson KM, Sherwood WG, Divry P, Rolland MO, Elpeleg ON, Rinaldo P, Jakobs C (1993). Reliable prenatal diagnosis of Canavan disease (aspartoacylase deficiency): comparison of enzymatic and metabolite analysis. J Inherit Metab Dis.

[278] Matalon R, Michals-Matalon K (1999). Prenatal diagnosis of canavan disease. Prenat Diagn.

[279] Matalon R, Michals K, Gashkoff P, Kaul R (1992). Prenatal diagnosis of canavan disease. J Inherit Metab Dis.

[280] Rolland MO, Divry P, Mandon G, Thoulon JM, Fiumara A, Mathieu M (1993). First-trimester prenatal diagnosis of Canavan disease. J Inherit Metab Dis.

[281] Rolland MO, Mandon G, Bernard A, Zabot MT, Mathieu M (1994). Unreliable verification of prenatal diagnosis of Canavan disease: aspartoacylase activity in deficient and normal fetal skin fibroblasts. J Inherit Metab Dis.

[282] Besley GTN, Elpeleg ON, Shaag A, Manning NJ, Jakobs C, Walter JH (1999). Prenatal diagnosis of Canavan disease—problems and dilemmas. J Inher Metab Dis.

[283] Elpeleg ON, Shaag A, Anikster Y, Jakobs C (1994). Prenatal detection of Canavan disease (aspartoacylase deficiency) by DNA analysis. J Inherit Metab Dis.

[284] Matalon R, Kaul R, Gao GP, Michals K, Gray RG, Bennett-Briton S, Norman A, Smith M, Jakobs C (1995). Prenatal diagnosis for Canavan disease: the use of DNA markers. J Inherit Metab Dis.

[285] Niu S, Ma Y, Lyu Y, Xin H, Wang D, Wang Y, Yang Y, Li Z, Liu Y, Gai Z (2024). Clinical and genetic analysis of a child with Canavan disease due to compound heterozygous variants of ASPA gene, Zhonghua yi xue yi chuan xue za zhi = Zhonghua yixue yichuanxue zazhi = Chinese. J Med Genet.

[286] Loeber JG, Burgard P, Cornel MC, Rigter T, Weinreich SS, Rupp K, Hoffmann GF, Vittozzi L (2012). Newborn screening programmes in Europe; arguments and efforts regarding harmonization part 1 from blood spot to screening result. J Inherit Metab Dis.

[287] Goldberg JD, Pierson S, Johansen Taber K (2023). Expanded carrier screening: What conditions should we screen for?. Prenat Diagn.

[288] Lund A, Wibrand F, Skogstrand K, Cohen A, Christensen M, Jäpelt RB, Dunø M, Skovby F, Nørgaard-Pedersen B, Gregersen N, Andresen BS, Olsen RKJ, Hougaard D (2020). Danish expanded newborn screening is a successful preventive public health programme. Dan Med J.

[289] Feigenbaum A, Moore R, Clarke J, Hewson S, Chitayat D, Ray PN, Stockley TL (2004). Canavan disease: carrier-frequency determination in the Ashkenazi Jewish population and development of a novel molecular diagnostic assay. Am J Med Genet A.

[290] Fares F, Badarneh K, Abosaleh M, Harari-Shaham A, Diukman R, David M (2008). Carrier frequency of autosomal-recessive disorders in the Ashkenazi Jewish population: should the rationale for mutation choice for screening be reevaluated?. Prenat Diagn.

[291] Klugman S, Gross SJ (2010). Ashkenazi Jewish screening in the twenty-first century. Obstet Gynecol Clin North Am.

[292] Kronn D, Oddoux C, Phillips J, Ostrer H (1995). Prevalence of Canavan disease heterozygotes in the New York metropolitan Ashkenazi Jewish population. Am J Hum Genet.

[293] Gross SJ, Pletcher BA, Monaghan KG (2008). Carrier screening in individuals of Ashkenazi Jewish descent. Genet Med.

[294] Sugarman EA, Allitto BA (2001). Carrier testing for seven diseases common in the Ashkenazi Jewish population: implications for counseling and testing. Obstet Gynecol.

[295] Strom CM, Crossley B, Redman JB, Quan F, Buller A, McGinniss MJ, Sun W (2004). Molecular screening for diseases frequent in Ashkenazi Jews: Lessons learned from more than 100,000 tests performed in a commercial laboratory. Genet Med.

[296] Zhang B, Dearing L, Amos J (2004). DNA-based carrier screening in the Ashkenazi Jewish population. Expert Rev Mol Diagn.

[297] Zayed H (2015). Canavan disease: an arab scenario. Gene.

[298] Kotambail A, Selvam P, Muthusamy K, Thomas M, Sudhakar SV, Ghati C, Danda S, Arunachal G (2023). Clustering of Juvenile Canavan disease in an Indian community due to population bottleneck and isolation: genomic signatures of a founder event. Eur J Hum Genet.

[299] Zeng B-J, Pastores GM, Leone P, Raghavan S, Wang Z-H, Ribeiro LA, Torres P, Ong E, Kolodny EH, Moffett JR, Tieman SB, Weinberger DR, Coyle JT, Namboodiri AMA (2006). Mutation analysis of the aspartoacylase gene in non-jewish patients with canavan disease. N-Acetylaspartate.

[300] Ashrafi M, Tavasoli A, Katibeh P, Aryani O, Vafaee-Shahi M (2015). A novel mutation in aspartoacylase gene, Canavan disease. Iran J Child Neurol.

[301] Sistermans EA, De Coo RFM, Van Beerendonk HM, Poll-The BT, Kleijer WJ, Van Oost BA (2000). Mutation detection in the aspartoacylase gene in 17 patients with Canavan disease: four new mutations in the non-Jewish population. Eur J Hum Genet.

[302] Pérez-Palma E, Gramm M, Nürnberg P, May P, Lal D (2019). Simple ClinVar: an interactive web server to explore and retrieve gene and disease variants aggregated in ClinVar database. Nucleic Acids Res.

[303] Manolio TA, Fowler DM, Starita LM, Haendel MA, MacArthur DG, Biesecker LG, Worthey E, Chisholm RL, Green ED, Jacob HJ, McLeod HL, Roden D, Rodriguez LL, Williams MS, Cooper GM, Cox NJ, Herman GE, Kingsmore S, Lo C, Lutz C, MacRae CA, Nussbaum RL, Ordovas JM, Ramos EM, Robinson PN, Rubinstein WS, Seidman C, Stranger BE, Wang H, Westerfield M, Bult C (2017). Bedside back to bench: building bridges between basic and clinical genomic research. Cell.

[304] Starita LM, Ahituv N, Dunham MJ, Kitzman JO, Roth FP, Seelig G, Shendure J, Fowler DM (2017). Variant interpretation: functional Assays to the Rescue. Am J Hum Genet.

[305] Landrum MJ, Chitipiralla S, Brown GR, Chen C, Gu B, Hart J, Hoffman D, Jang W, Kaur K, Liu C, Lyoshin V, Maddipatla Z, Maiti R, Mitchell J, O’Leary N, Riley GR, Shi W, Zhou G, Schneider V, Maglott D, Holmes JB, Kattman BL (2020). ClinVar: improvements to accessing data. Nucleic Acids Res.

[306] Karczewski KJ, Francioli LC, Tiao G, Cummings BB, Alföldi J, Wang Q, Collins RL, Laricchia KM, Ganna A, Birnbaum DP, Gauthier LD, Brand H, Solomonson M, Watts NA, Rhodes D, Singer-Berk M, England EM, Seaby EG, Kosmicki JA, Walters RK, Tashman K, Farjoun Y, Banks E, Poterba T, Wang A, Seed C, Whiffin N, Chong JX, Samocha KE, Pierce-Hoffman E, Zappala Z, O’Donnell-Luria AH, Minikel EV, Weisburd B, Lek M, Ware JS, Vittal C, Armean IM, Bergelson L, Cibulskis K, Connolly KM, Covarrubias M, Donnelly S, Ferriera S, Gabriel S, Gentry J, Gupta N, Jeandet T, Kaplan D, Llanwarne C, Munshi R, Novod S, Petrillo N, Roazen D, Ruano-Rubio V, Saltzman A, Schleicher M, Soto J, Tibbetts K, Tolonen C, Wade G, Talkowski ME, Aguilar Salinas CA, Ahmad T, Albert CM, Ardissino D, Atzmon G, Barnard J, Beaugerie L, Benjamin EJ, Boehnke M, Bonnycastle LL, Bottinger EP, Bowden DW, Bown MJ, Chambers JC, Chan JC, Chasman D, Cho J, Chung MK, Cohen B, Correa A, Dabelea D, Daly MJ, Darbar D, Duggirala R, Dupuis J, Ellinor PT, Elosua R, Erdmann J, Esko T, Färkkilä M, Florez J, Franke A, Getz G, Glaser B, Glatt SJ, Goldstein D, Gonzalez C, Groop L, Haiman C, Hanis C, Harms M, Hiltunen M, Holi MM, Hultman CM, Kallela M, Kaprio J, Kathiresan S, Kim B-J, Kim YJ, Kirov G, Kooner J, Koskinen S, Krumholz HM, Kugathasan S, Kwak SH, Laakso M, Lehtimäki T, Loos RJF, Lubitz SA, Ma RCW, MacArthur DG, Marrugat J, Mattila KM, McCarroll S, McCarthy MI, McGovern D, McPherson R, Meigs JB, Melander O, Metspalu A, Neale BM, Nilsson PM, O’Donovan MC, Ongur D, Orozco L, Owen MJ, Palmer CNA, Palotie A, Park KS, Pato C, Pulver AE, Rahman N, Remes AM, Rioux JD, Ripatti S, Roden DM, Saleheen D, Salomaa V, Samani NJ, Scharf J, Schunkert H, Shoemaker MB, Sklar P, Soininen H, Sokol H, Spector T, Sullivan PF, Suvisaari J, Tai ES, Teo YY, Tiinamaija T, Tsuang M, Turner D, Tusie-Luna T, Vartiainen E, Vawter MP, Ware JS, Watkins H, Weersma RK, Wessman M, Wilson JG, Xavier RJ, Neale BM, Daly MJ, MacArthur DG (2020). The mutational constraint spectrum quantified from variation in 141,456 humans. Nature.

[307] Gersing S, Schulze TK, Cagiada M, Stein A, Roth FP, Lindorff-Larsen K, Hartmann-Petersen R (2023). Characterizing glucokinase variant mechanisms using a multiplexed abundance assay. BioRxiv Prepr Serv Biol.

[308] Gersing S, Cagiada M, Gebbia M, Gjesing AP, Coté AG, Seesankar G, Li R, Tabet D, Weile J, Stein A, Gloyn AL, Hansen T, Roth FP, Lindorff-Larsen K, Hartmann-Petersen R (2023). A comprehensive map of human glucokinase variant activity. Genome Biol.

[309] Jia X, Burugula BB, Chen V, Lemons RM, Jayakody S, Maksutova M, Kitzman JO (2021). Massively parallel functional testing of MSH2 missense variants conferring Lynch syndrome risk. Am J Hum Genet.

[310] Scott A, Hernandez F, Chamberlin A, Smith C, Karam R, Kitzman JO (2022). Saturation-scale functional evidence supports clinical variant interpretation in lynch syndrome. Genome Biol.

[311] Matreyek KA, Starita LM, Stephany JJ, Martin B, Chiasson MA, Gray VE, Kircher M, Khechaduri A, Dines JN, Hause RJ, Bhatia S, Evans WE, Relling MV, Yang W, Shendure J, Fowler DM (2018). Multiplex assessment of protein variant abundance by massively parallel sequencing. Nat Genet.

[312] Frazer J, Notin P, Dias M, Gomez A, Min JK, Brock K, Gal Y, Marks DS (2021). Disease variant prediction with deep generative models of evolutionary data. Nature.

[313] Laine E, Karami Y, Carbone A (2019). GEMME: a simple and fast global epistatic model predicting mutational effects. Mol Biol Evol.

[314] Brandes N, Goldman G, Wang CH, Ye CJ, Ntranos V (2022). Genome-wide prediction of disease variants with a deep protein language model. BioRxiv.

[315] Cheng J, Novati G, Pan J, Bycroft C, Žemgulytė A, Applebaum T, Pritzel A, Wong LH, Zielinski M, Sargeant T, Schneider RG, Senior AW, Jumper J, Hassabis D, Kohli P, Avsec Ž (2023). Accurate proteome-wide missense variant effect prediction with alphamissense. Science.

[316] Livesey BJ, Marsh JA (2022). Interpreting protein variant effects with computational predictors and deep mutational scanning. DisModel Mech.

[317] Ng PC, Henikoff S (2001). Predicting deleterious amino acid substitutions. Genome Res.

[318] Schymkowitz J, Borg J, Stricher F, Nys R, Rousseau F, Serrano L (2005). The FoldX web server: an online force field. Nucleic Acids Res.

[319] Park H, Bradley P, Greisen P, Liu Y, Mulligan VK, Kim DE, Baker D, Dimaio F (2016). Simultaneous optimization of biomolecular energy functions on features from small molecules and macromolecules. J Chem Theory Comput.

[320] Cagiada M, Bottaro S, Lindemose S, Schenstrøm SM, Stein A, Hartmann-Petersen R, Lindorff-Larsen K (2023). Discovering functionally important sites in proteins. Nat Commun.

[321] Cagiada M, Johansson KE, Valanciute A, Nielsen SV, Hartmann-Petersen R, Yang JJ, Fowler DM, Stein A, Lindorff-Larsen K (2021). Understanding the origins of loss of protein function by analyzing the effects of thousands of variants on activity and abundance. Mol Biol Evol.

[322] Tsuboyama K, Dauparas J, Chen J, Laine E, Mohseni Behbahani Y, Weinstein JJ, Mangan NM, Ovchinnikov S, Rocklin GJ (2023). Mega-scale experimental analysis of protein folding stability in biology and design. Nature.

[323] Kots ED, Khrenova MG, Nemukhin AV (2019). Allosteric control of N-Acetyl-aspartate hydrolysis by the Y231C and F295S mutants of human aspartoacylase. J Chem Inf Model.

[324] Krishnamoorthy N, Zayed H (2016). Structural modeling of p.V31F variant in the aspartoacylase gene. Metab Brain Dis.

[325] Zaki OK, Krishnamoorthy N, El Abd HS, Harche SA, Mattar RA, Al Disi RS, Nofal MY, El Bekay R, Ahmed KA, George Priya Doss C, Zayed H (2017). Two patients with Canavan disease and structural modeling of a novel mutation metab. Brain Dis.

[326] George Priya Doss C, Zayed H (2017). Comparative computational assessment of the pathogenicity of mutations in the aspartoacylase enzyme metab. Brain Dis.

[327] Guex N, Peitsch MC (1997). SWISS-MODEL and the Swiss-PdbViewer: an environment for comparative protein modeling. Electrophoresis.

[328] Salentin S, Schreiber S, Haupt VJ, Adasme MF, Schroeder M (2015). PLIP: fully automated protein-ligand interaction profiler. Nucleic Acids Res.

[329] Chen C-W, Lin J, Chu Y-W (2013). iStable: off-the-shelf predictor integration for predicting protein stability changes. BMC Bioinform.

[330] Glaser F, Pupko T, Paz I, Bell RE, Bechor-Shental D, Martz E, Ben-Tal N (2003). ConSurf: identification of functional regions in proteins by surface-mapping of phylogenetic information. Bioinformatics.

[331] Pucci F, Schwersensky M, Rooman M (2022). Artificial intelligence challenges for predicting the impact of mutations on protein stability. Curr Opin Struct Biol.

[332] David A, Sternberg MJE (2023). Protein structure-based evaluation of missense variants: resources, challenges and future directions. Curr Opin Struct Biol.

[333] Aradhya S, Facio FM, Metz H, Manders T, Colavin A, Kobayashi Y, Nykamp K, Johnson B, Nussbaum RL (2023). Applications of artificial intelligence in clinical laboratory genomics. Am J Med Genet C Semin Med Genet.

[334] Kampmeyer C, Nielsen SV, Clausen L, Stein A, Gerdes A-M, Lindorff-Larsen K, Hartmann-Petersen R (2017). Blocking protein quality control to counter hereditary cancers., genes. chromosomes. Cancer.

[335] Stein A, Fowler DM, Hartmann-Petersen R, Lindorff-Larsen K (2019). Biophysical and mechanistic models for disease-causing protein variants. Trends Biochem Sci.

[336] Arora K, Naren AP (2016). Pharmacological correction of cystic fibrosis: molecular mechanisms at the plasma membrane to augment mutant CFTR function. Curr Drug Targets.

[337] Kile BT, Hentges KE, Clark AT, Nakamura H, Salinger AP, Liu B, Box N, Stockton DW, Johnson RL, Behringer RR, Bradley A, Justice MJ (2003). Functional genetic analysis of mouse chromosome 11. Nature.

